# Appraising the Genetic Architecture of Kernel Traits in Hexaploid Wheat Using GWAS

**DOI:** 10.3390/ijms21165649

**Published:** 2020-08-06

**Authors:** Ali Muhammad, Weicheng Hu, Zhaoyang Li, Jianguo Li, Guosheng Xie, Jibin Wang, Lingqiang Wang

**Affiliations:** 1 College of Plant Science and Technology & Biomass and Bioenergy Research Center, Huazhong Agricultural University, Wuhan 430070, China; alikhaksar87@webmail.hzau.edu.cn (A.M.); huweichen@webmail.hzau.edu.cn (W.H.); chaoyangli@gmail.com (Z.L.); ooozil0325@gmail.com (J.L.); xiegsh@mail.hzau.edu.cn (G.X.); 2 State Key Laboratory for Conservation and Utilization of Subtropical Agro-Bioresources, College of Agriculture, Guangxi University, 100 Daxue Rd., Nanning 530004, China

**Keywords:** wheat, kernel traits, genome-wide association study, single nucleotide polymorphism

## Abstract

Kernel morphology is one of the major yield traits of wheat, the genetic architecture of which is always important in crop breeding. In this study, we performed a genome-wide association study (GWAS) to appraise the genetic architecture of the kernel traits of 319 wheat accessions using 22,905 single nucleotide polymorphism (SNP) markers from a wheat 90K SNP array. As a result, 111 and 104 significant SNPs for Kernel traits were detected using four multi-locus GWAS models (mrMLM, FASTmrMLM, FASTmrEMMA, and pLARmEB) and three single-locus models (FarmCPU, MLM, and MLMM), respectively. Among the 111 SNPs detected by the multi-locus models, 24 SNPs were simultaneously detected across multiple models, including seven for kernel length, six for kernel width, six for kernels per spike, and five for thousand kernel weight. Interestingly, the five most stable SNPs (RAC875_29540_391, Kukri_07961_503, tplb0034e07_1581, BS00074341_51, and BobWhite_049_3064) were simultaneously detected by at least three multi-locus models. Integrating these newly developed multi-locus GWAS models to unravel the genetic architecture of kernel traits, the mrMLM approach detected the maximum number of SNPs. Furthermore, a total of 41 putative candidate genes were predicted to likely be involved in the genetic architecture underlining kernel traits. These findings can facilitate a better understanding of the complex genetic mechanisms of kernel traits and may lead to the genetic improvement of grain yield in wheat.

## 1. Introduction

Bread wheat (*Triticum aestivum* L.) is a staple food crop worldwide, providing 20% of the caloric intake of humans [1,2]. Food production needs to be increase by an estimated 50% in order to keep pace with the increasing demands projected for 2050. This requires continuing genetic gains in yield improvement by approximately 1.1% per annum [3]. Enhancing the yield by evaluating grain traits is a common target for genetic improvement in wheat breeding. Grain yield is governed by multiple genes, which are greatly influenced by environmental factors [4]. The total grain yield depends on multiple yield components, such as grain number per spike, thousand kernel weight, kernel length, and kernel width, where each component is quantitatively inherited [5].

Grain morphology has been studied for decades, but determining loci associated with seed dimensions remains an important task in crop yield improvement studies [6]. This has led to the search for fundamental genes associated with grain weight and size, with several genes controlling grain morphology having been identified recently [7]. Among these genes, *TaGW2* has been associated with grain morphology, which is an orthologous gene of rice *OsGW2* [8]. Several quantitative trait loci (QTLs) in the promoter region of *TaGW2-6A* associated with kernel weight and kernel dimension parameters have been identified [8,9,10]. Heidari et al. [11] and Zhang et al. [12] identified several QTLs associated with kernel number per spike (KPS). Using a set of wheat cultivars, Guo et al. [13,14] screened 13 KPS-associated QTLs. Although there have been several studies related to QTL mapping and genome-wide association studies (GWASs) for yield-related traits [15,16,17,18,19], only a few outcomes have been applied for wheat improvement in breeding programs [20]. High-throughput single nucleotide polymorphism (SNP) assays have been used in breeding and genotyping studies, which enable the process of association mapping to be more accurate and robust [20,21]. SNP assays developed from animal and plant transcriptomes [22] are generally more advanced, stable, capable of automatic detection, efficient, and cost-effective [23,24]. In wheat, several high-density SNP arrays (i.e., 9K [24], 90K [22], 820K [25], and 660K [26], among others) have been adopted. Among these, the 90K and 660K arrays are now widely applied, as compared to simple sequence repeat (SSR) markers, for the genetic improvement of wheat traits related to yield, quality, stress tolerance, and disease resistance [27,28,29,30]. Recently, the 90K array based on the Illumina platform can be found in bi-parental QTL mapping [31,32,33,34] and GWAS for yield-contributing traits in wheat [35,36,37]. Conventional QTL mapping can only reflect the genetic content between parents and focuses on specific traits [20]. Recent advances in genomic technologies have shown GWAS to be a promising alternative approach to QTL mapping. Recently, several genomic regions associated with yield-related traits have been identified through genome-wide association studies (GWASs) in wheat [20,38,39,40].

GWAS relies on linkage disequilibrium (LD), which dissects the genetic basis of complex traits. Unlike QTL mapping, GWAS can explore wider genetic variations with higher resolution, in order to phenotype several traits with one cycle of genotyping [20]. It is an effective method for detecting multi-allelic variation, thus helping in the pyramiding of favorable alleles for a target trait [41]. Commonly used GWAS approaches utilizing mixed linear models have become the most standard criteria, employing population structure or kinship analysis among genotypes [42]. However, due to the strengthened screening criteria of Bonferroni correction, many important small-effect loci cannot be detected, especially with a large number of SNPs [43]. In order to obtain the associations at a fine mapping resolution, subsequent mixed-model applications have integrated multiple methods with a higher detection power. Multi-locus models fit large-effect loci as covariates, allowing for the identification of more loci with smaller effects [44]. Recently, several multi-locus models, including mrMLM [45], FASTmrEMMA [46], pLARmEB [47], and FASTmrMLM [48] have been developed, with high powers of detection and less strong criteria.

Therefore, the present study was designed to conduct GWAS in a set of 319 wheat accessions in central China and the Yangzi River region, using the high-throughput wheat 90K iSelect SNP array [22]. The objectives of this study included: (1) the detection of significant loci associated with kernel traits, (2) comparing and integrating the effects of loci on kernel traits, and (3) identifying the candidate genes which control the corresponding kernel traits. Finally, the detection of several significant SNPs by integrating three single-locus and four recently developed multi-locus GWAS models can facilitate marker-assisted breeding for higher yields in wheat.

## 2. Results

### 2.1. Phenotypic Evaluation, Correlation, and Heritability of Kernel Traits

**Figure 1:**
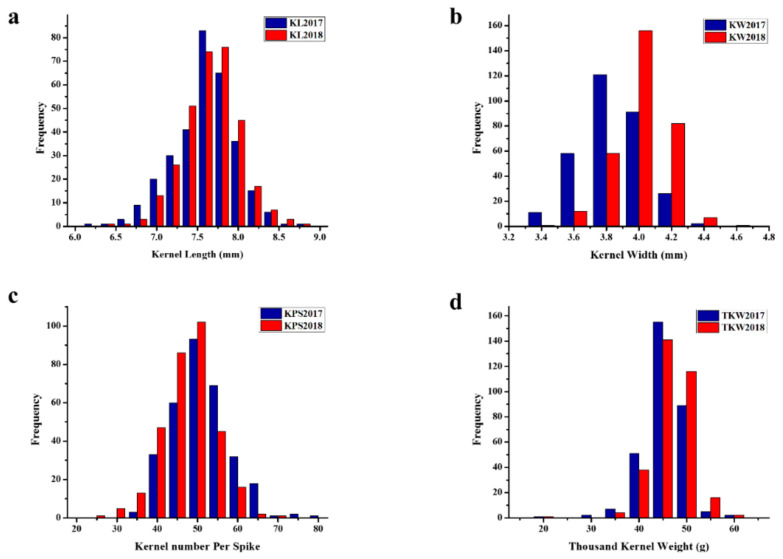
Frequency distribution of the kernel traits: (**a**) the kernel length, (**b**) kernel width, (**c**) kernels per spike, and (**d**) the thousand kernel weight in 2017 and 2018.

Phenotypic variations of kernel traits among the 319 wheat accessions were evaluated at the experimental field in Wuhan during the winter seasons of 2017 and 2018. Phenotypic evaluation revealed significant variations for all traits (i.e., kernel length, kernel width, kernels per spike, and thousand kernel weight) in the two years (Table S1). Kernel length ranged from 6.36 mm to 8.62 mm with a mean of 7.53 mm, where the data revealed a normal distribution pattern with a standard deviation of 0.34 and coefficient of variation (CV) of 4.53%. Kernel width ranged from 2.89 mm to 4.37 mm with a CV of 4.58%. For kernels per spike, the number of kernels in each spike ranged from 28.08 to 68.93. Thousand kernel weight ranged from 17.98 g to 56.99 g. The frequency distributions of all traits revealed a normal distribution in each year, as presented in Figure 1, indicating the quantitative nature of these traits.

**Figure 2:**
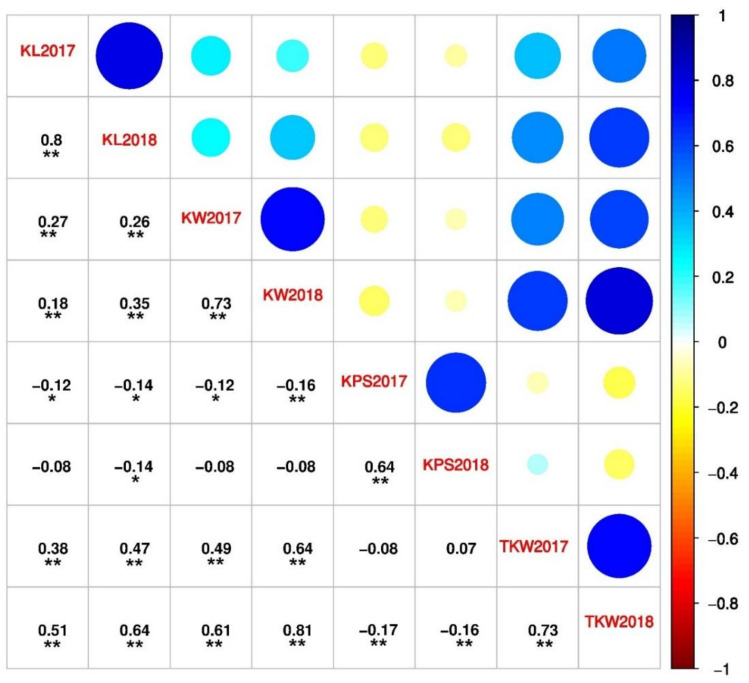
Correlation between the kernel traits, including KL (kernel length), KW (kernel width), KPS (kernels per spike), and TKW (thousand kernel weight) for 2017 and 2018. Significant correlations are shown with asterisk (*). * and **, significant at *p <* 0.05 and *p <* 0.01, respectively.

The correlation analysis showed significant positive correlations between the two years for kernel length (r = 0.80), kernel width (r = 0.73), kernels per spike (r = 0.63), and thousand kernel weight (r = 0.73), indicating the consistency of these traits across the years. Furthermore, the correlation coefficients showed a significant positive association (*p* < 0.01) of thousand kernel weight with both kernel width (r = 0.49–0.81) and length (r = 0.38–0.64), indicating that the kernel weight was influenced by kernel size. By contrast, kernel length and width showed significant negative correlations with the kernels per spike (Figure 2). The ANOVA results revealed significant differences (*p* < 0.01) among the genotypes for all traits (Table S2). High broad-sense heritability was observed for all traits, with a range from 0.80 to 0.89, indicating that these traits were largely influenced by genetic factors.

### 2.2. Population Structure and LD Analysis

Population structure analysis is mandatory in a diverse population, as the presence of a large number of genotypes in a study can result in a higher quantity of false associations between the phenotypes and unlinked markers. Hence, a detailed examination of the population structure is necessary for conducting a valid association analysis. The number of sub-populations were determined by the changing value of the log probability of data between the successive *K*-values. Δ*K* was estimated by the STRUCTURE analysis, in order to highlight the increase in the *K* value, following the procedure of Evanno [49]. From the kinship analysis, a break in the slope was observed at *K* = 2, followed by a flattening of the curve (Figure S1a); hence, it was revealed that the most likely number of sub-populations was two (*K* = 2; Figure S1b). Furthermore, these results were confirmed by principle component analysis (PCA), based on the standardized covariance of genetic distances of SNP markers (Figure S1c). Linkage disequilibrium (LD) among markers was performed using TASSEL v.5.0. software, indicating the mapping resolution and power. The LD among SNPs was estimated for the whole genome (Figure S2a). Only markers with a known position and with an MAF greater than 5% were used. The LD half-width i.e., the point at which LD decays to 50% of the peak, was 9.8 cM. With an increase in genetic distance, the *r^2^* value of the A, B, and D sub-genomes decreased gradually (Figure S2b). The LD analysis for sub-genomes revealed the highest marker density on B (58%) followed by A (34.6%) and then D (7.4%) sub-genomes. Chromosome 2B has the highest marker density, while 4D has the lowest. There was a high LD for most of the pairwise comparisons between the SNP loci. The presence of population structure was one reason inferred for the high LD. The two main factors that greatly affected the significant LD between the independent loci were removing similar entries and considering the presence of population structure. Overall, the LD decays not very rapidly which reveals the significance of the markers used in the study.

### 2.3. GWAS for Kernel Traits

**Figure 3:**
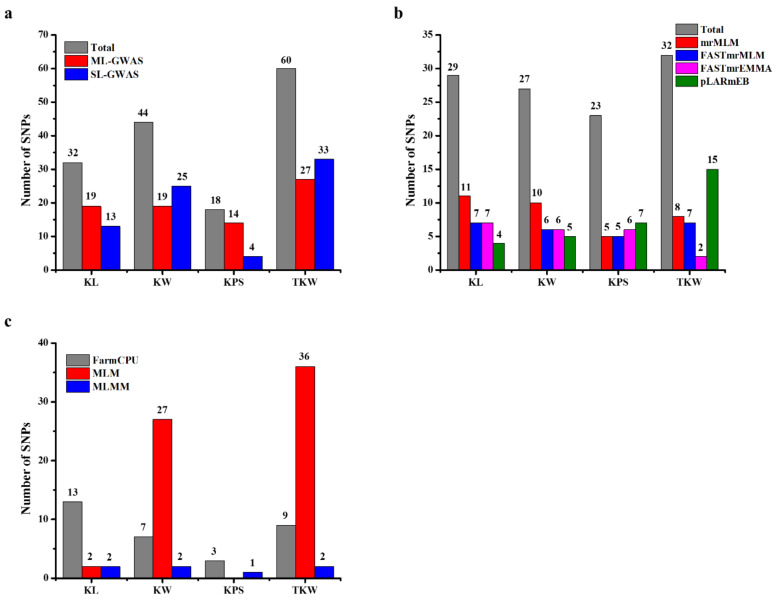
Significant single nucleotide polymorphisms (SNPs) detected by different multi-locus genome-wide association study (ML-GWAS) and single-locus GWAS (SL-GWAS) methods for the Kernel traits of the Kernel length, Kernel width, Kernels per spike, and thousand kernel weight: (**a**) Number of detected SNPs through ML-GWAS and SL-GWAS methods; (**b**) number of significant SNPs detected by ML-GWAS methods including mrMLM, FASTmrMLM, FASTmrEMMA, and pLARmEB; and (**c**) significant SNPs detected by SL-GWAS methods including FarmCPU, MLM, and MLMM.

We conducted the GWAS to unravel the complex genetic architecture of kernel traits, including kernel length, kernel width, kernels per spike, and thousand kernel weight, across two environments. Four multi-locus GWAS (ML-GWAS)—mrMLM, FASTmrMLM, FASTmrEMMA, and pLARmEB—and three single-locus GWAS (SL-GWAS) methods—FarmCPU, MLM, and MLMM—were used to detect significant loci associated with these traits. In ML-GWAS, loci with critical logarithm of odds (LOD) values equal to 3 or greater than 3 were considered significant. For SL-GWAS, the selection criteria were set to *p* = 1*/n* (*n* corresponding to the total number of SNPs). To obtain more reliable results, the SNPs which were simultaneously detected by at least two methods or in two years were regarded as consensus SNPs. By not considering the repeated SNPs by different models, we obtained a total of 79 and 75 loci for the kernel traits detected on all 21 chromosomes using the ML-GWAS and SL-GWAS methods, respectively (Figure 3a).

### 2.4. Loci Detected by Multi-Locus GWAS Methods

Four multi-locus GWAS methods in the mrMLM software were used in this study. A total of 34, 25, 21, and 31 significant SNPs were detected by mrMLM, FASTmrMLM, FASTmrEMMA, and pLARmEB, respectively, for KL (29), KW (27), KPS (23), and TKW (32) across two environments (Figure 3b). Among these SNPs, 11, 10, five, and eight were detected by mrMLM for KL, KW, KPS, and TKW, respectively. By using FASTmrMLM, a total of seven, six, five, and seven were detected associated with the aforementioned objective traits, respectively. Furthermore, seven, six, six, and two significant loci were identified by FASTmeEMMA for these traits, respectively. Finally, by applying the pLARmEB ML-GWAS approach, a total of four, five, seven, and 15 significant SNPs were associated with KL, KW, KPS, and TKW, respectively (Figure 3b).

**Figure 4:**
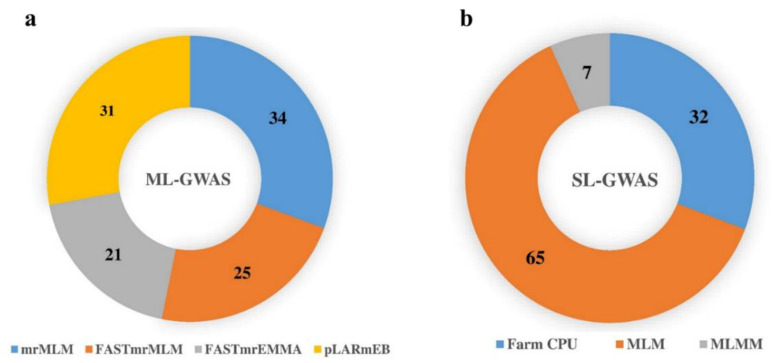
Significant SNPs detected by ML-GWAS vs. SL-GWAS methods: (**a**) the total number of significant SNPs detected by four ML-GWAs methods (mrMLM, FASTmrMLM, FASTmrEMMA, and pLARmEB); and (**b**) the total number of significant SNPs detected by three SL-GWAS methods (FarmCPU, MLM, and MLMM).

**Figure 5:**
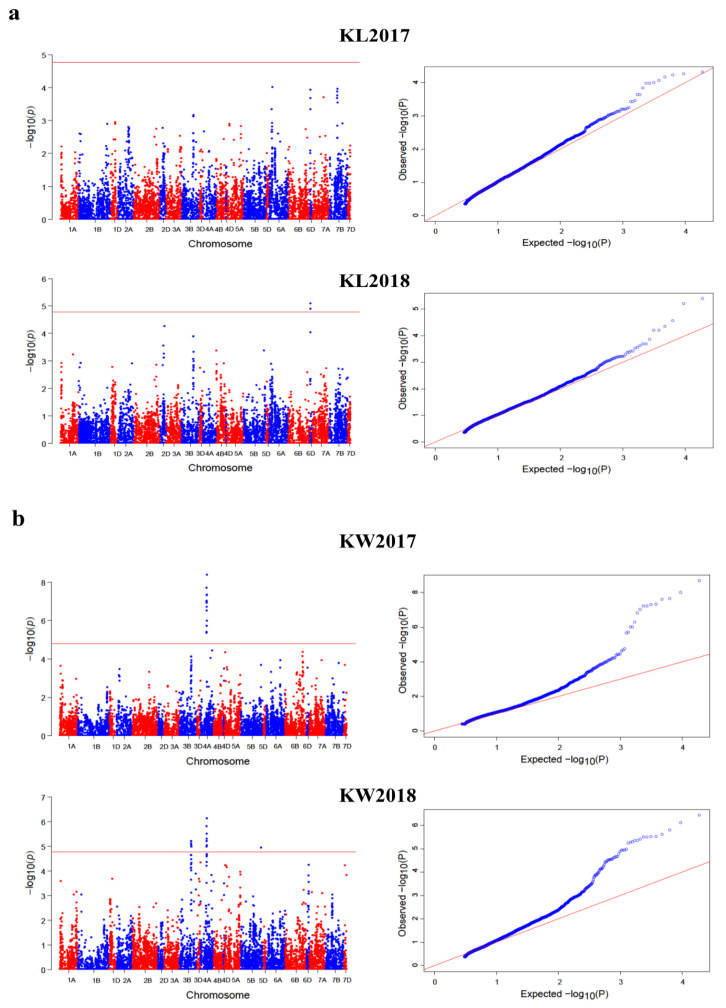

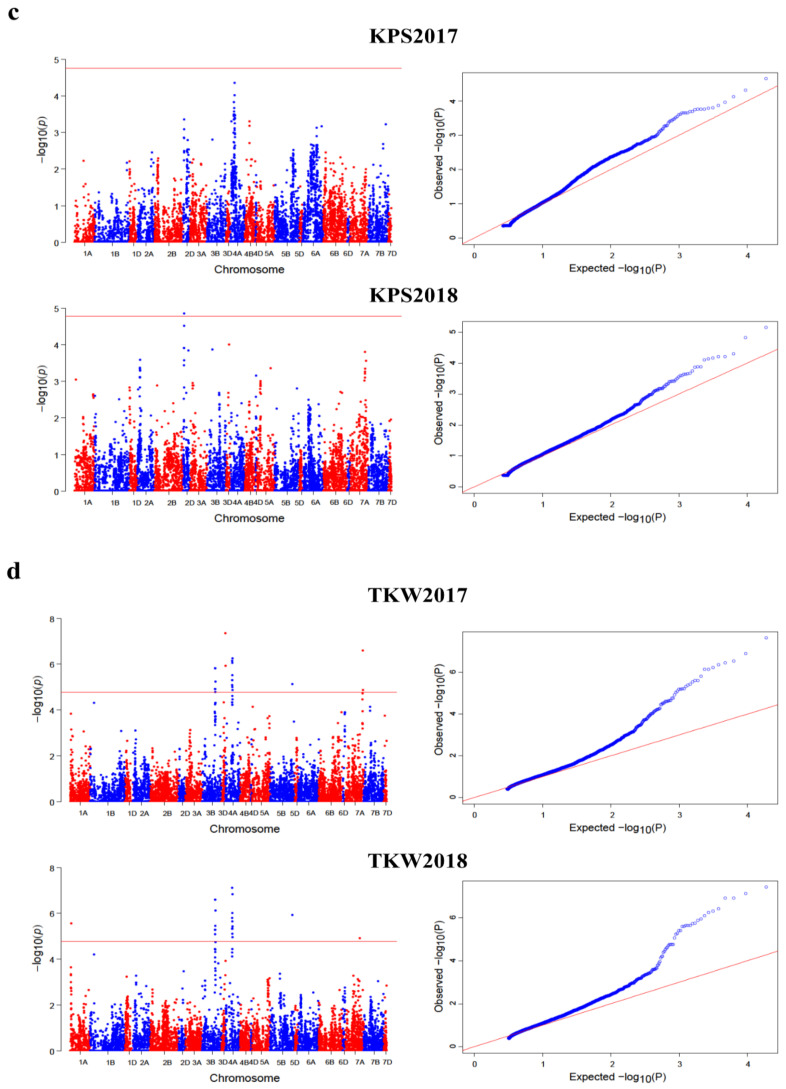
Manhattan and quantile–quantile (Q–Q) plots for the kernel traits across two environments (2017–2018): (**a**) Manhattan and Q–Q plots for kernel length; (**b**) Manhattan and Q–Q plots for kernel width; (**c**) Manhattan and Q–Q plots for kernels per spike; and (**d**) Manhattan and Q–Q plots for thousand kernel weight. Note: Number of detected SNPs by four multi-locus GWAS methods. For brevity, we present here the Manhattan and Q–Q plots of mrMLM model. The rest of the Manhattan and Q–Q plots are given in Supplementary Figure S3.

For further validation, we compared the results across the different ML-GWAS models and both years. As a result, we found 24 significant SNPs co-detected by at least two ML-GWAS methods, whereas three SNPs were consistently repeated in both years. Interestingly, most of these SNPs were located on chromosome 7A and 7B, while chromosome 1B harbored only one SNP. Among the 24 stably expressed SNPs across different ML-GWAS models, five SNPs (RAC875_29540_391, Kukri_07961_503, tplb0034e07_1581, BS00074341_51, and BobWhite_049_3064) were simultaneously detected by at least three ML-GWAS methods. Furthermore, to observe the association of these consistent SNPs, seven loci were associated with KL with phenotypic variance (PVE) explained by the SNPs ranging from 2.58% to 10.51%. The remaining consistent SNPs associated with the rest of kernel traits are shown in Table 1. By integrating the four ML-GWAS methods (mrMLM, FASTmrMLM, FASTmrEMMA, and pLARmEB) to appraise the genetic architecture of the kernel traits, the mrMLM models detected the most SNPs (34), most of which contributed to KL (11 SNPs), while FASTmrEMMA detected the least SNPs (21; Figure 4a). Among the significant SNPs common to both years, two SNPs (Ku_008899_90, Kukri_07961_503) were detected for KL, with an LOD ranging from 3.64 to 7.50, while one SNP (BS00021738_51) was detected across both years for KW, with an LOD score from 4.98 to 7.30 (Table 1). Manhattan plots showing the distribution of significant SNPs for KL, KW, KPS, and TKW, as well as the Q–Q plots illustrating the observed vs. expected associations, are given in Figure 5.

### 2.5. Loci Detected by Single-Locus GWAS Methods

Single-locus GWAS (SL-GWAS) models, including FarmCPU, MLM, and MLMM, were implemented using the Genomic association and prediction integrated tool (GAPIT) package in R, in order to further evaluate the above kernel traits. A total of 75 significant SNPs was detected by integrating the three SL-GWAS methods in two environments (Figure 3c; Table S3). Among these SNPs, chromosome 4A harbored the maximum number of SNPs (27), followed by chromosome 3B (12 SNPs) and chromosome 7A (five SNPs). By comparing the results of the three SL-GWAS methods, FarmCPU detected 32, MLM identified 65, and MLMM detected seven significant loci associated with kernel traits across the two environments. After observing the association of these SNPs with different kernel traits, we found a total of 17 SNPs associated with KL, 36 SNPs with KW, four SNPs with KPS, and 47 SNPs with TKW (Figure 3c). We further analyzed the SNPs commonly detected by all three SL-GWAS methods and highlighted seven SNPs which were simultaneously detected by more than one SL-GWAS method. Among these SNPs, Kukri_07961_503 was simultaneously detected by all three SL-GWAS methods (Table S4). Conclusively, the MLM approach detected the maximum number of SNPs (65)—mostly contributing to TKW—while MLMM detected the least SNPs (seven; Figure 4b). To obtain more reliable results, we integrated the results of both ML-GWAS and SL-GWAS methods and detected a total of 13 SNPs common to both ML-GWAS and SL-GWAS methods (Table 2). Among these SNPs, most were detected on chromosomes 4A and 7A, thus revealing the importance of these chromosomes in the wheat genome. After observing the associations of these SNPs, we found that most of these SNPs were associated with TKW, followed by KL. Based on these findings, it can be concluded that these SNPs are more reliable and stable, which may be useful for further breeding purposes. Manhattan and Q–Q plots of the above three single-locus GWAS models for plant architectural traits are given in Supplementary Figure S4.

### 2.6. SNPs Associated with More than One Trait

SNPs controlling more than one trait are prominent in marker-assisted selection. In the present study, we screened some candidate SNPs through the ML-GWAS methods and detected a total of five pleiotropic SNPs (Excalibur_01167_1207, RAC875_00174_268, D_contig07330_330, IACX938, and Tdurum_contig15734_221) associated with different kernel traits (Table S5). Among these, three SNPs (Excalibur_01167_1207, RAC875_00174_268, and D_contig07330_330), located on chromosomes 5A, 5B, and 7D, respectively, were associated with KW and TKW. However, one SNP (IACX938), which was located on chromosome 4B, controlled KL and KW. Finally, one SNP (Tdurum_contig15734_221), located on chromosome 7B, was associated with KL, KW, and TKW at the same time. As discussed above, most of the SNPs had common associations with KW and TKW, which was also confirmed by the correlation analysis (Figure 2). Based on these findings, it can be concluded that these pleotropic SNPs have multifaceted roles in controlling the genetic architecture of kernel traits.

### 2.7. Mining of Candidate Genes

**Figure 6:**
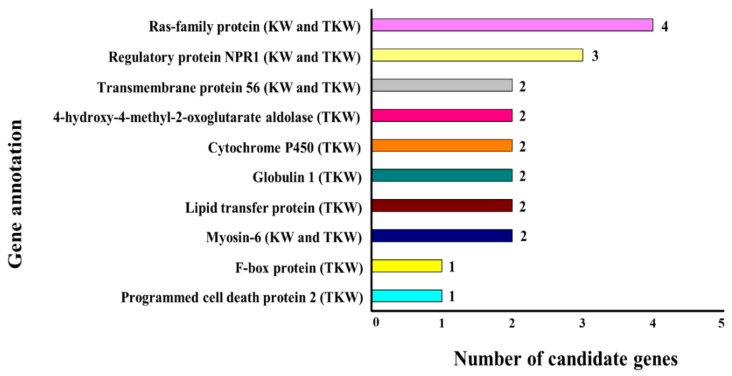
Functional classification of putative candidate genes controlling the kernel width and the thousand kernel weight. The number of SNPs (for either single or multiple traits) located within the genes that have the same gene annotations is shown. KW, kernel width; TKW, thousand kernel weight.

Candidate genes were detected around the peak SNPs, based on the annotated wheat reference genome. A total of 41 putative candidate genes were associated with kernel traits (Table S6). Among these, six genes located on chromosomes 4A and 3B were associated with kernel width, while the other 35 genes (which were mostly located on chromosomes 3B, 4A, 5B, and 7A) had putative roles in controlling the thousand kernel weight. Most of the candidate genes were located on chromosome 4A followed by chromosome 7A. Based on these results, the genes were categorized regarding several important functions associated with plant growth and development (including Ras family protein, Regulatory protein NPR1, Myosin 6, and so on), as shown in Figure 6 and Table S6.

## 3. Discussion

### 3.1. Phenotypic Observations of Kernel Traits

Wheat (*Triticum aestivum* L.) is one of the major staple food crops, which has an important role in achieving food security [50]. Therefore, improving kernel yield and related traits are consistently important tasks for genetic gain in this crop. Kernel size and shape are key quantitative characteristics which influence crop yield and end-use quality in wheat. Kernel dimensions in wheat have been studied for decades, but the genes underlying kernel morphology are poorly understood [6]. Kernel traits—particularly kernel length, kernel width, kernels per spike, and thousand kernel weight—are key components of kernel yield and quality, due to their significant roles in cultivar yield, milling quality, and market price [51]. The present study was undertaken to use four multi-locus GWAS (ML-GWAS) methods—mrMLM, FASTmrMLM, FASTmrEMMA, and pLARmEB—and three single-locus GWAS (SL-GWAS) methods—FarmCPU, MLM, and MLMM—to detect the significant loci associated with kernel traits in a collection of 319 wheat accessions with a broad genetic base.

Manual measurements of kernel traits have limited data quantity and quality. Therefore, data analysis using digital image technology can improve the process of measurement while providing robust and quick results [52]. Several studies have been done to unravel kernel morphology in wheat by using digital image technologies [6,52,53,54,55,56,57]. The phenotypic traits showed significant variations for kernel traits with high heritability. The significant positive correlation of kernel width and length with kernel weight revealed that the kernels with increased width and length can greatly contribute to kernel weight. However, in the current study, kernel width had a more positive impact on kernel weight than kernel length. Earlier studies have reported moderate to strong correlations between kernel weight and size [56]. Simmonds et al. [58] reported that kernel length and width in tetraploid and hexaploid wheat can greatly influence the thousand kernel weight, as longer and broader kernels have more starch accumulation and hence, a higher weight. Similarly, the presence of a positive and significant correlation between the kernel length and kernel width suggests that choosing heavier kernels may result in an indirect selection for larger seeds. Previously, Breseghello and Sorrells [54] and Ramya et al. [59] reported positive associations among kernel weight, kernel length, and kernel width.

### 3.2. GWAS Using High-Throughput Genotyping

The advent of next-generation sequencing technologies has enabled the detection of many SNPs, demonstrating high-density genotyping compared to conventional marker analysis [22]. SNP chips are highly adapted, due to their stability, power of detection, efficiency, and low cost [22,29]. At present, molecular studies in wheat using SNP chips are still at a preliminary level. For instance, [60] evaluated 102 Argentinean hexaploid wheat varieties using a 35K SNP array to detect ninety-seven genomic regions associated with yield-contributing traits, and further screened fifteen markers controlling the fruiting efficiency at harvest (FEh), which is an important trait to increase wheat yield. Talini et al. [61] characterized a collection of 299 *Triticum Urartu* accessions and developed 441,327 single nucleotide polymorphisms to detect the significant loci associated with several agronomic and quality traits. Furthermore, they revealed that *T. Urartu* may provide an important allele pool for wheat improvement and detected 25 significant quantitative trait nucleotides (QTNs) associated with the different traits under study. Wheat strip rust is one of the most serious treats to wheat yield. Therefore, genetic resistance against this disease is the most effective strategy to control the disease. Cheng et al. [62] conducted GWAS in 120 China winter wheat accessions using SNP markers of a 90K array and detected 16 significant loci controlling wheat strip rust. Gahlaut, Jaiswal, Singh, Balyan, and Gupta [38] conducted multi-locus GWAS in a collection of 320 spring wheat accessions using 9626 SNPs and detected 46 drought-tolerant loci associated with different traits. Chen et al. [63] conducted a metabolite-based GWAS study using 14,646 SNP markers and detected 1098 genomic regions and 42 candidate genes having prominent effects.

### 3.3. Comparison of Present GWAS Results with Previous Studies

Recently, several studies based on 90K SNP arrays can be found which have used GWAS for kernel yield [20,64,65], for resistance against different diseases in wheat [62,66,67], and for bi-parental QTL mapping [32,68,69,70,71]. Comparatively, GWAS-based linkage disequilibrium (LD) is a much more powerful tool to detect genetic loci in larger gene pools, with much higher resolution than traditional QTL mapping [72]. GWAS can be used to phenotype several complex traits in studies with larger population sizes [20]. The validity of GWAS results depends on the number of markers used in the study. In the present study, we used 22,905 SNPs from a wheat 90K array to detect several genomic loci for kernel traits. A total of 24 common SNPs was detected by the different ML-GWAS methods. However, we only found three SNPs which were consistently repeated in both years. We note that Zhang et al. [73] detected only one common QTN in three environments in a total of 129 QTNs identified for seed protein content in soybean. Su et al. [74] detected a total of 70 significant SNPs for fiber-related traits in upland cotton, of which only three SNPs were found across two environments. We believe that the inconsistency between the two years is mostly due to the Kernel yield and its contributing traits possessing a complex quantitative nature, representing the culmination of a diverse array of various vegetative and reproductive processes [75]. In addition, we detected moderate interaction effects between the accessions (genotypes) and the years. Furthermore, the correlation between the two years does not definitely indicate an association in the genetics. Due to the inconsistency of these SNPs across environments, we focused on common SNPs across different GWAS models, as the major purpose of this study was to highlight the most accurate SNPs. Therefore, we considered the results of all four ML-GWAS methods and selected the SNPs identified by multiple methods as credible SNPs to consider in further experiments. Most of these SNPs were located on chromosome 7A and 7B. Jamil et al. [76] conducted GWAS for seven agronomic traits and revealed the importance of chromosome 7A, due to the presence of association signals with several agronomic traits. According to Wang et al. [77], most of the MTAs for plant height- and yield-contributing traits were located on chromosome 7B, suggesting the importance of this chromosome. Furthermore, several previous reports have indicated the prominence of chromosome 7B by revealing many yield-related genes and QTLs on chromosome 7B, including *Hkps/sn-7B* loci for increased grains and spikelet number per spike [78], the *TaSus1-7B* genomic region for TKW [79], and the *TaCYP78A3* candidate gene for grain size [80]. According to our results, common SNPs detected by different ML-GWAS models associated with KW were located on chromosomes 2A, 4A, 6A, 7B, and 7D, where chromosome 2A was more promising for KW, harboring most of the stable SNPs. Similarly, the SNPs linked to KW on 2A have been reported earlier in genome-wide association studies for grain yield in hexaploid wheat [20] and in QTL mapping for seed dimensions in bread wheat [6].

By further reviewing the results of commonly identified SNPs in the ML-GWAS methods, a stable SNP (Tdurum_contig15734_221) was consistently repeated by three ML-GWAS methods, which we referred to as a pleiotropic SNP, having association signals with multiple traits identified for kernel length and width, on chromosome 7B. The same SNP has been identified in a bi-parental QTL mapping for plant height- and yield-contributing traits in hexaploid wheat [81]. Another pleiotropic SNP (D_contig07330_330) associated with kernel width and thousand kernel weight was identified in our study, which was located on chromosome 7D. A similar SNP has been reported earlier, in a genome-wide association study for domestication and the improvement of candidate loci using a 90K wheat SNP array [82]. Another stable SNP (tplb0034e07_1581) associated with kernel width was detected in our study, which was consistently repeated by three ML-GWAS methods. Previously, Yang et al. [83] reported the same SNP by evaluating disease resistance genes in wheat and Arabidopsis. 

Kernel number per spike is another important trait for kernel yield in wheat. For KPS, most of the stable SNPs were located on chromosome 7A, as revealed by the ML-GWAS results. Earlier studies conducted on GWAS for yield-contributing traits reported significant loci linked to KPS on chromosome 7A [20,77]. A stable SNP (Tdurum_contig54559_211) associated with KPS was simultaneously detected by two ML-GWAS methods (mrMLM and pLARmEB). Zanke et al. [84] reported the same SNP in a genome-wide study for thousand kernel weight in hexaploid wheat. TKW is a complex quantitative trait, which had SNPs located on different chromosomes, including 4A, 7A, and 7D. Most of the SNPs for TKW were located on 7A. Similarly, SNPs for TKW located on chromosome 7A have been previously reported in several studies [76,77,85]. In our study, we retrieved high-confidence candidate genes surrounding (±200 kb) the peak SNPs, based on the annotated wheat reference genome IWGSC RefSeq v1.0 [86]. A total of 41 putative genes were detected and categorized into major functional groups (Figure 6). These functional groups included the “Ras family protein”, controlling TKW and KW. Ras family proteins are involved in the virulence and the cell death of harmful fungi [87,88]. Regulatory protein NPR1 is another functional protein associated with KW and TKW. NPR1 plays a prominent role in developing systematic acquired resistance (SAR) [89]. According to Diethelm et al. [90], NPR1-like genes are involved in Fusarium head blight (FHB) resistance in wheat. Another functional protein included putative Cytochrome P450, which is involved in plant defense, including resistance to FHB disease in wheat [91,92]. Furthermore, Globulin 1 was associated with TKW, which is a major class of storage proteins involved in seed development [93]. Other functional proteins were predicted for KW and TKW, including putative transmembrane protein 56, Lipid transfer protein, Myosin-6, and so on, as shown in Supplementary Table S6.

## 4. Materials and Methods 

### 4.1. Plant Materials

The plant material was comprised of 319 wheat accessions obtained from the Hubei Academy of Agricultural Sciences in Hubei Province, China, which represent a wheat gene pool adapted to the central China and the Yangzi River region. Most of the accessions comprised of released varieties and landraces (266), some of which were genetic stocks (41) and improved genotypes (12). These accessions were evaluated in randomized complete blocks with three replicates at the experimental farm of Huazhong Agricultural University (HZAU), Wuhan, China, for two consecutive winter seasons (2017–2018 and 2018–2019). Twenty individual plants from each accession were selected and grown in two rows, with distances of 15 cm between the plants and 20 cm between the rows. Field management essentially followed normal local wheat cropping practices. The plants were harvested individually at maturity for phenotype evaluation.

### 4.2. Phenotyping

Four traits, including kernel length (KL), kernel width (KW), kernels per spike (KPS), and thousand kernel weight (TKW), were evaluated at the experimental farm of HZAU, Wuhan, China, during the winter seasons of 2017 and 2018. The measurements of these kernel traits were performed by selecting the main spikes of five random individual plants in the middle of the row for each accession. Kernels per spike were estimated by hand-threshing the mature spike. Thousand kernel weight (TKW) of each plant was recorded by weighing all the seeds from a sample, dividing it by the total seed number measured, and multiplying the result by 1000 [6]. In the experiment, we counted the number of seeds from each sample, with more than 200 seeds. Phenotypic analysis was done by high-throughput image processing, as follows: the kernels were placed on a glass plate and images were produced by a scanner (GT-X820, Epson, Suwa, Japan) at a resolution of 240 or 360 dpi. The images were processed using the software package *SmartGrain* ver. 1.2, developed for the high-throughput phenotyping of rice grains [94], and which can also be used for wheat grain measurement [95]. Measurements were done with the default threshold for the accuracy of grain detection, such that extreme values for grain size could be excluded.

### 4.3. Genotyping

The 319 wheat accessions were genotyped using the Illumina 90K wheat chip [22] in the genotyping Laboratory of North Dakota State University in Fargo. Quality pre-processing of the genotyping data was performed using the PLINK software (https://zzz.bwh.harvard.edu/plink/) [96] for the sample call rate, SNP call rate, minor allele frequency (MAF), and Hardy–Weinberg equilibrium (HWE).

### 4.4. Statistical Analysis

Statistical analyses, including ANOVA, heritability, correlation, and descriptive statistics, were conducted using the R statistical package [97]. The broad-sense heritability for the traits was estimated by the formula *H*^2^ = VG/(VG + VE) where VG and VE represent the estimates of genetic and environmental variance, respectively [75].

### 4.5. Population Structure and Kinship Analysis

Population structure analysis was carried out by the STRUCTURE 2.3.4 software [98], using Bayesian cluster analysis, and the obtained results were visualized with the STRUCTURE HARVESTER software [99], in order to obtain an appropriate K value. The putative number of sub-populations (*k* = 1 to 7) was assessed using 100,000 burn-in iterations followed by 500,000 recorded Markov chain iterations. In order to reveal the sampling variance (robustness) of the inferred population structure, 10 independent runs were carried out for each K value. K was estimated, according to the method of Evanno et al. [49], using an ad hoc statistic, ∆K, based on the rate of change in the log probability of data between successive values. Principle component analysis (PCA) was calculated by R software to evaluate the population structure and for comparison with the results of STRUCTURE [29]. Linkage disequilibrium (LD) among the markers was also performed using the observed vs. expected allele frequencies of the markers in the TASSEL v.5.0 software [64].

### 4.6. Genome-Wide Association Studies

To appraise the genetic architecture of kernel-related traits, we used mrMLM software for four multi-locus GWAS (ML-GWAS), including FASTmrMLM, FASTmrEMMA, mrMLM, and pLARmEB, as well as three single-locus GWAS (SL-GWAS) models, including FarmCPU, MLM, and MLMM, as implemented by the Genomic Association and Prediction Integrated Tool (GAPIT) in R [100]. Previously, GLM and MLM were the most generally applied among SL-GWAS methods. However, these methods have some limitations; for example, GLM can encounter high false-positive rates (FPRs), while MLM uses Bonferroni corrections to reduce FPRs [101] and so this method is so stringent that it may ignore several significant SNPs [45]. To overcome these issues, multi-locus GWAS approaches are the best alternatives. The strict selection criterion in the SL-GWAS analysis was substituted by a flexible selection criterion in multi-locus GWAS analysis, which lessened the possibility of missing out significant loci [45,46]. The four ML-GWAS methods were performed with default parameters, while the screening criteria for significance were set with the logarithm of odds (LOD) scores of 3 or greater than 3 [43,45,46,47,102]. However, for the SL-GWAS models, the threshold for the *p*-value was calculated based on the number of markers (*p* = 1*/n*, *n* = total SNPs used), according to the method of [103]. Significant markers were visualized with a Manhattan plot using the Haploview 4.2 software [104]. Important *p*-value distributions (expected vs. observed *p*-values on a -log^10^ scale) are shown with a quantile–quantile plot.

### 4.7. Candidate Gene Analysis

The R Package Pathway Association Study Tool (PAST) version 1.0.1 [105] was used to detect high-confidence genes surrounding (±200 kb) the peak SNPs. Gene sites were aligned and downloaded from the ViroBLAST database (https://urgi.versailles.inra.fr/blast/docs/aboutviroblast.html). To obtain the putative related candidate genes of SNP-flanking regions, a BLASTx search was conducted for significant marker-trait associations (MTAs) against the recently released genome sequence i.e. international wheat genome sequencing consortium (IWGSC) RefSeq v1.0 [86].

## 5. Conclusions

The present study utilized four multi-locus GWAS (ML-GWAS) and three single-locus GWAS (SL-GWAS) models, resulting in the detection of the consensus SNPs and genes responsible for the determination of kernel traits. Comparatively, 111 and 104 significant SNPs were found to be significant using the ML-GWAS and SL-GWAS methods, respectively. By integrating these GWAS models, the mrMLM and MLM approaches outperformed in SNP detection compared to the other ML-GWAS and SL-GWAS models, respectively. Overall, the ML-GWAS results revealed five stable SNPs (RAC875_29540_391, Kukri_07961_503, tplb0034e07_1581, BS00074341_51, and BobWhite_049_3064), which were simultaneously detected by at least three of the ML-GWAS methods. Furthermore, 41 putative candidate genes were assessed as controlling kernel width and thousand kernel weight, thus providing new insights into the genetic architecture of these kernel traits.

## Figures and Tables

**Table 1:** Common SNPs identified by different multi-locus models for kernel traits (kernel length kernel width, kernels per spike, and thousand kernel weight).

Trait	Methods (1–4)	SNPs	Chr	Position (bp)	LOD > 3	r^2^ (%)	Repeated in Years
KL	1,2,3	RAC875_29540_391	1A	264	4.44–6.42	3.41–4.84	2018
	2,3	BS00089524_51	1B	637	3.07–5.99	4.64–6.57	2018
	1,2,4	Kukri_07961_503	6D	3,491,885	6.14–7.50	3.63–7.65	2017, 2018
	1,2	Kukri_c8827_217	7A	3,732,575	3.56–3.74	2.70–4.40	2017
	1,4	Tdurum_contig102328_129	7B	4,030,937	3.94–6.58	5.36–10.51	2017
	1,3	Kukri_c64387_218	7B	4,049,359	3.22–4.07	2.58–4.84	2018
	1,2	Tdurum_contig15734_221	7B	4,127,623	3.60–4.59	2.64–4.33	2018
	1	Ku_008899_90	3D	1,664,604	3.64–3.86	5.22–5.40	2017, 2018
KW	1,2	RAC875_0643_1548	2A	105,090	3.10–3.31	1.10–3.99	2017
	2,3,4	tplb0034e07_1581	2A	79,588	3.86–4.29	1.28–1.94	2018
	1,2	Kukri_c48194_641	4A	1,892,065	3.35–6.32	4.59–6.97	2017
	1,4	Ra_02239_504	6A	2,962,548	3.95–4.59	1.88–4.77	2018
	2,3	Tdurum_contig15734_221	7B	4,127,623	3.40–4.33	2.92–3.65	2017
	2,3	D_contig07330_330	7D	4,292,747	3.01–6.28	2.50–6.00	2017
	1	BS00021738_51	4A	1,804,501	4.98–7.30	14.34–16.13	2017, 2018
KPS	2,3,4	BS00074341_51	1A	109	4.18–6.18	2.90–3.96	2018
	1,4	Tdurum_contig54559_211	4B	2,056,791	3.27–4.03	2.16–4.47	2017
	2,4	Kukri_08268_79	5A	2,172,100	3.26–3.51	2.39–3.87	2018
	2,3	GENE_3726_78	6B	3,355,233	3.28–3.68	2.32–2.40	2018
	1,2,3,4	BobWhite_049_3064	7A	3,802,457	3.26–9.02	2.83–8.15	2018
	2,4	wsnp_Ku_rep_004159_90704469	7A	3,806,941	5.07–5.36	3.41–4.07	2018
TKW	2,4	wsnp_Ex_c33778_42210283	4A	1,954,125	5.99–7.90	1.21–6.84	2017
	1,4	RAC875_rep_c83934_91	7A	3,886,333	3.76–10.77	2.27–5.15	2017
	2,4	wsnp_Ku_0552_3060297	7A	3,779,727	3.83–4.82	2.24–3.50	2018
	1,2	Kukri_rep_c97425_164	7A	3,791,938	3.19–3.34	1.86–3.34	2018
	2,4	D_contig07330_330	7D	4,292,747	3.43–3.54	0.49–3.10	2017

Methods (1–4): the corresponding multi-locus GWAS methods (mrMLM, FASTmrMLM, FASTmrEMMA, and pLARmEB, respectively). r^2^ (%) represents the proportion of the total phenotypic variation explained by each SNP. KL (kernel length); KW (kernel width); KPS (kernels per spike); TKW (thousand kernel weight).

**Table 2:** Common SNPs simultaneously co-detected by multi-locus and single-locus GWAS methods for the kernel length, kernel width, kernels per spike, and the thousand kernel weight.

Trait	Methods	SNP	Chr	Position (bp)	Repeated in Years
KL	pLARmEB, MLMM	Kukri_07961_503	6D	3,491,885	2017, 2018
KL	mrMLM, FarmCPU	Kukri_c8827_217	7A	3,732,575	2017
KL	pLARmEB, FarmCPU	Tdurum_contig102328_129	7B	4,030,937	2017
KW	mrMLM, MLMM	BS00021738_51	4A	1,804,501	2017, 2018
KW	FASTmrMLM, FarmCPU	Tdurum_contig15734_221	7B	4,127,623	2017
KPS	mrMLM, MLMM	wsnp_Ra_rep_007017_90667618	4A	1,774,526	2017
KPS	mrMLM, FarmCPU	BobWhite_049_3064	7A	3,802,457	2018
TKW	FASTmrEMMA, FarmCPU	BobWhite_09733_301	1B	868	2017
TKW	mrMLM, MLMM	BS00110286_51	3D	1,727,487	2017
TKW	mrMLM, MLMM	Ex_07338_401	4A	1,800,847	2017, 2018
TKW	mrMLM, MLM	Kukri_rep_c97425_164	7A	3,791,938	2018
TKW	pLARmEB, MLM	RAC875_rep_c83934_91	7A	3,886,333	2017
TKW	FASTmrEMMA, MLM	wsnp_Ex_c4068_7351806	4A	1,803,131	2017, 2018

KL (kernel length); KW (kernel width); KPS (kernels per spike); TKW (thousand kernel weight).

## Supplementary Materials

The following are available online at https://www.mdpi.com/1422-0067/21/16/5649/s1, Figure S1: Structure analysis of 319 wheat accessions based on unlinked SNPs. Figure S2: Linkage disequilibrium (LD) analysis among markers. Figure S3: Manhattan and Q–Q plots of multi-locus GWAS models for kernel length, kernel width, kernels per spike, and thousand kernel weight in two years. Figure S4: Manhattan and Q–Q plots of three single-locus GWAS models for kernel traits in two years. Table S1: Phenotypic variation of kernel length, kernel width, kernel per spike, and thousand kernel weight in two years (2017–2018). Table S2: Analysis of variance (ANOVA) and heritability for kernel length, kernel width, kernel per spike, and thousand kernel weight. Table S3: Significant SNPs detected by three single-locus GWAS methods in two years for kernel length, kernel width, kernels per spike, and thousand kernel weight. Table S4: Common SNPs co-detected by multiple- and single-locus GWAS methods for kernel length, kernel width, and thousand kernel weight. Table S5: List of pleiotropic SNPs associated with more than one trait. Table S6: List of putative candidate genes around the peak SNPs associated with kernel width (KW) and thousand kernel weight (TKW).

## Abbreviations

GWAS	Genome-wide association study
ML	Multi-locus
SL	Single-locus
SNP	Single nucleotide polymorphism
QTL	Quantitative trait loci
KL	Kernel length
KW	Kernel width
KPS	Kernel per spike
TKW	Thousand kernel weight
MAF	Minor allele frequency
HWE	Hardy–Weinberg equilibrium
ANOVA	Analysis of variance
LD	Linkage disequilibrium
GAPIT	Genomic association and prediction integrated tool
MTA	Marker-trait associations
CV	Coefficient of variation
