# Supplementary material for: Appraising the Genetic Architecture of Kernel Traits in Hexaploid Wheat Using GWAS
Source: Int J Mol Sci. 2020 Aug 6;21(16):5649. doi: 10.3390/ijms21165649 (PMC7460857; doi:10.3390/ijms21165649)
Supplement: Supplementary file 1 [file ijms-21-05649-s001.zip › Supplementary data/Figure S1-S4.pptx]

## Slide 1
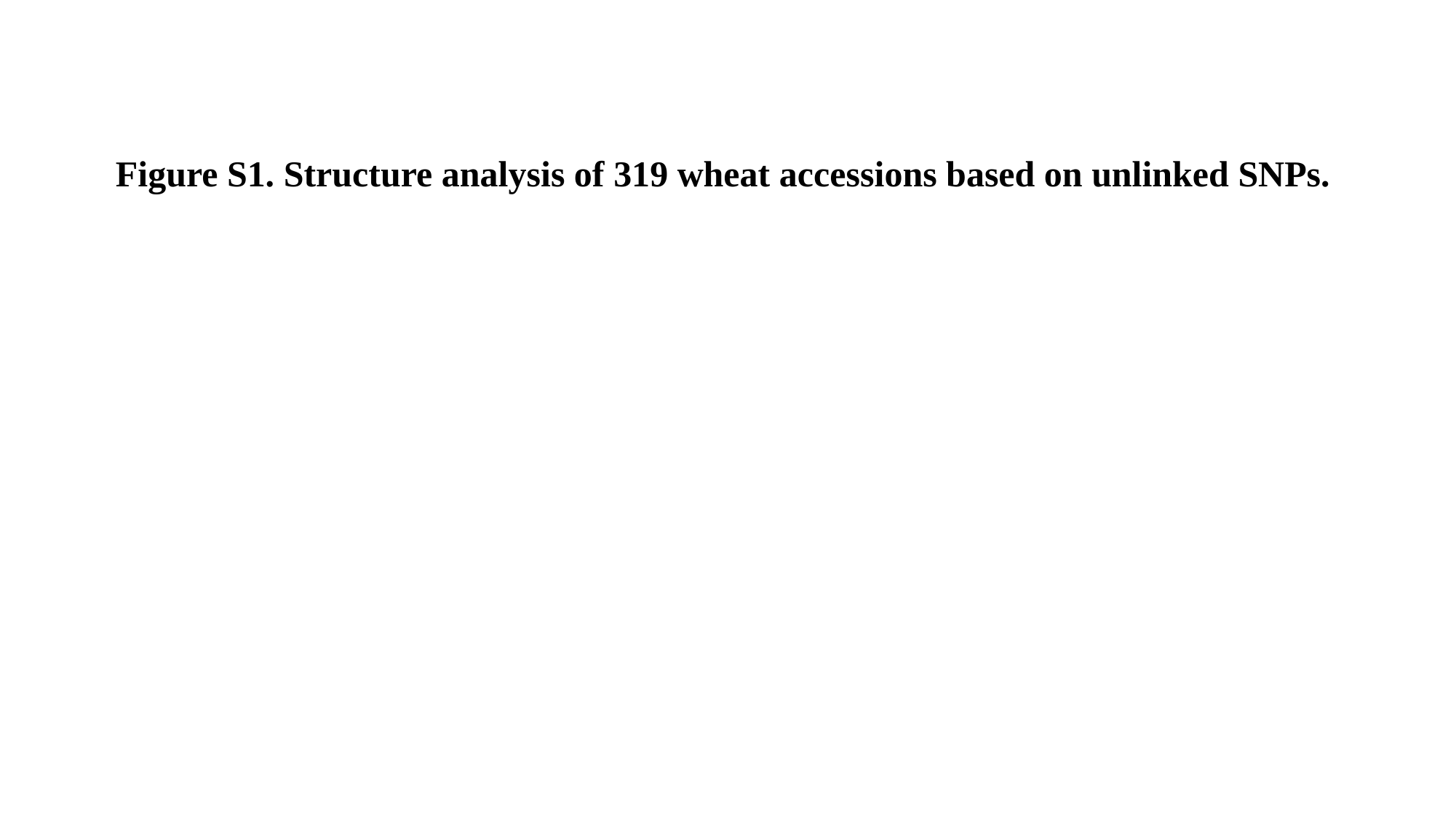

Figure S1. Structure analysis of 319 wheat accessions based on unlinked SNPs.

## Slide 2
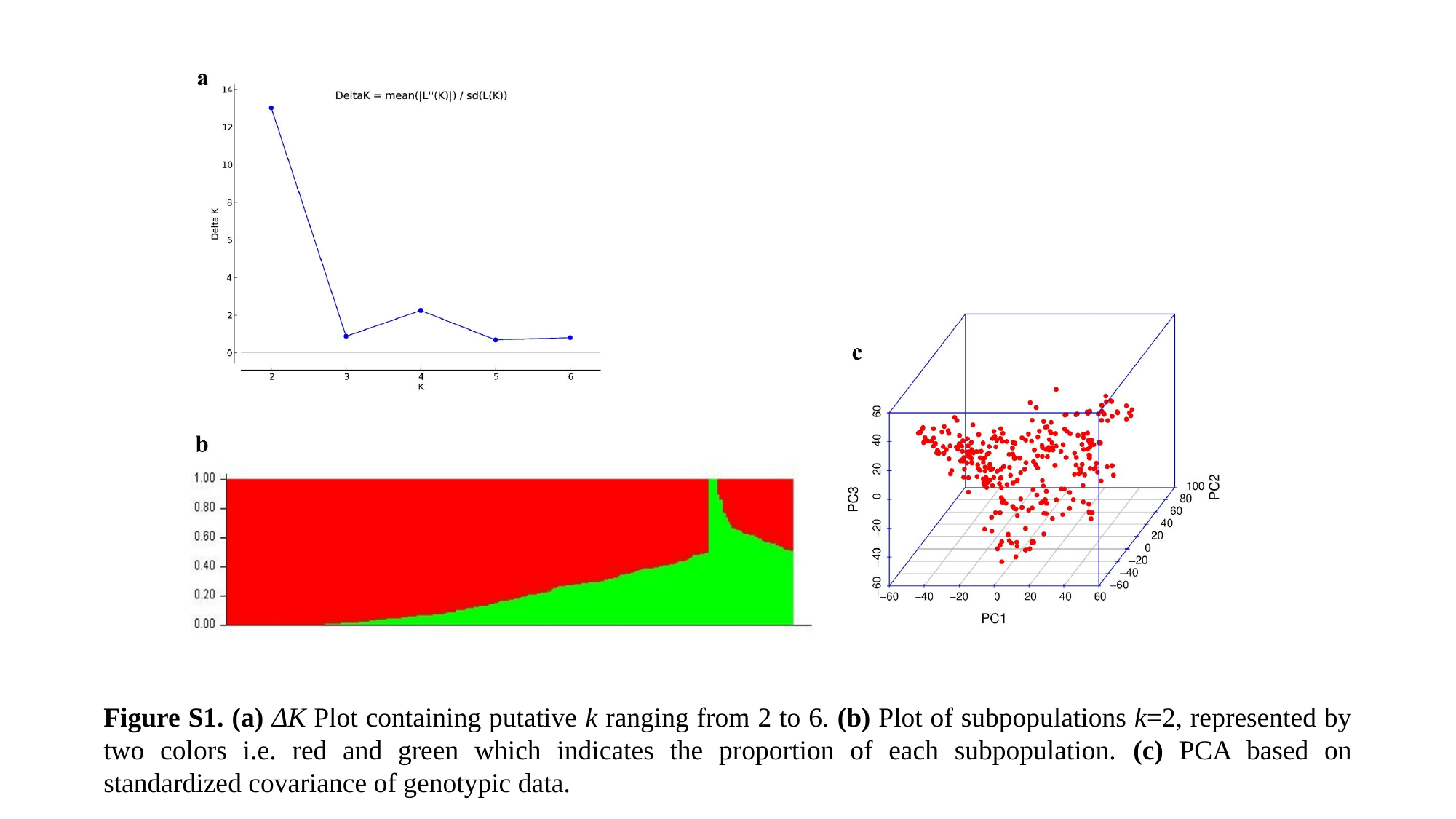

Figure S1. (a) ΔK Plot containing putative k ranging from 2 to 6. (b) Plot of subpopulations k=2, represented by two colors i.e. red and green which indicates the proportion of each subpopulation. (c) PCA based on standardized covariance of genotypic data.

## Slide 3
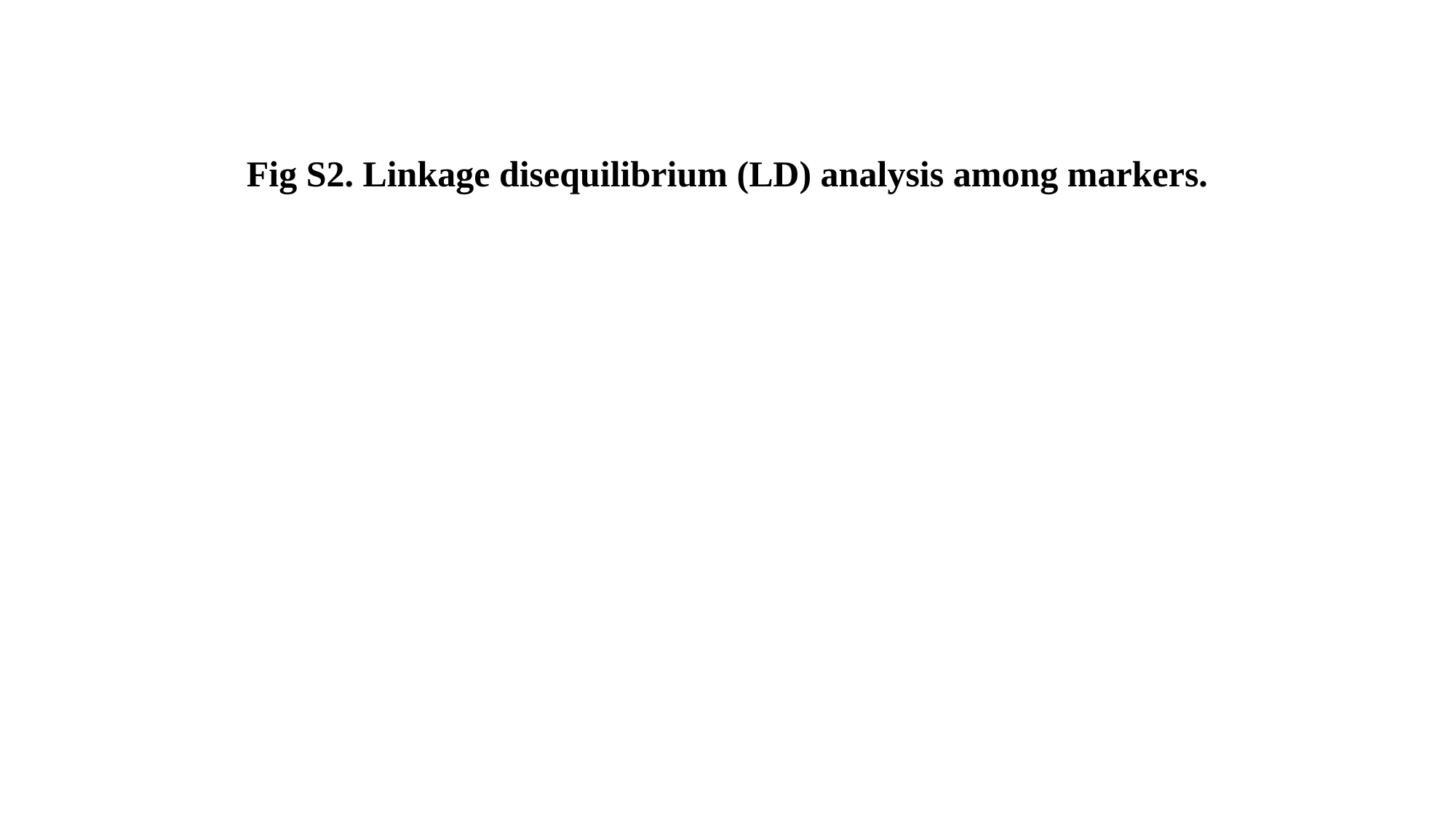

Fig S2. Linkage disequilibrium (LD) analysis among markers.

## Slide 4
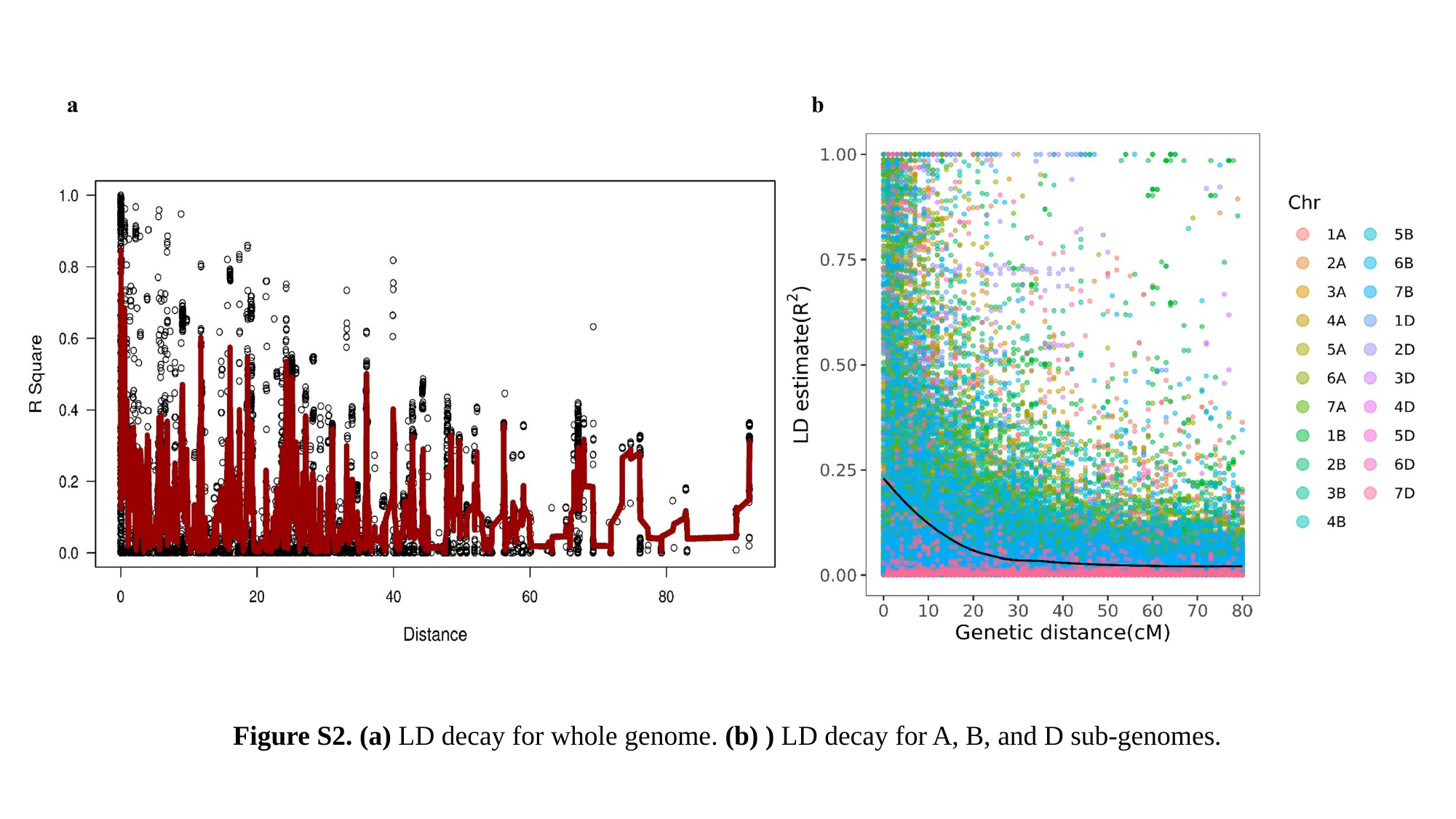

Figure S2. (a) LD decay for whole genome. (b) ) LD decay for A, B, and D sub-genomes.

## Slide 5
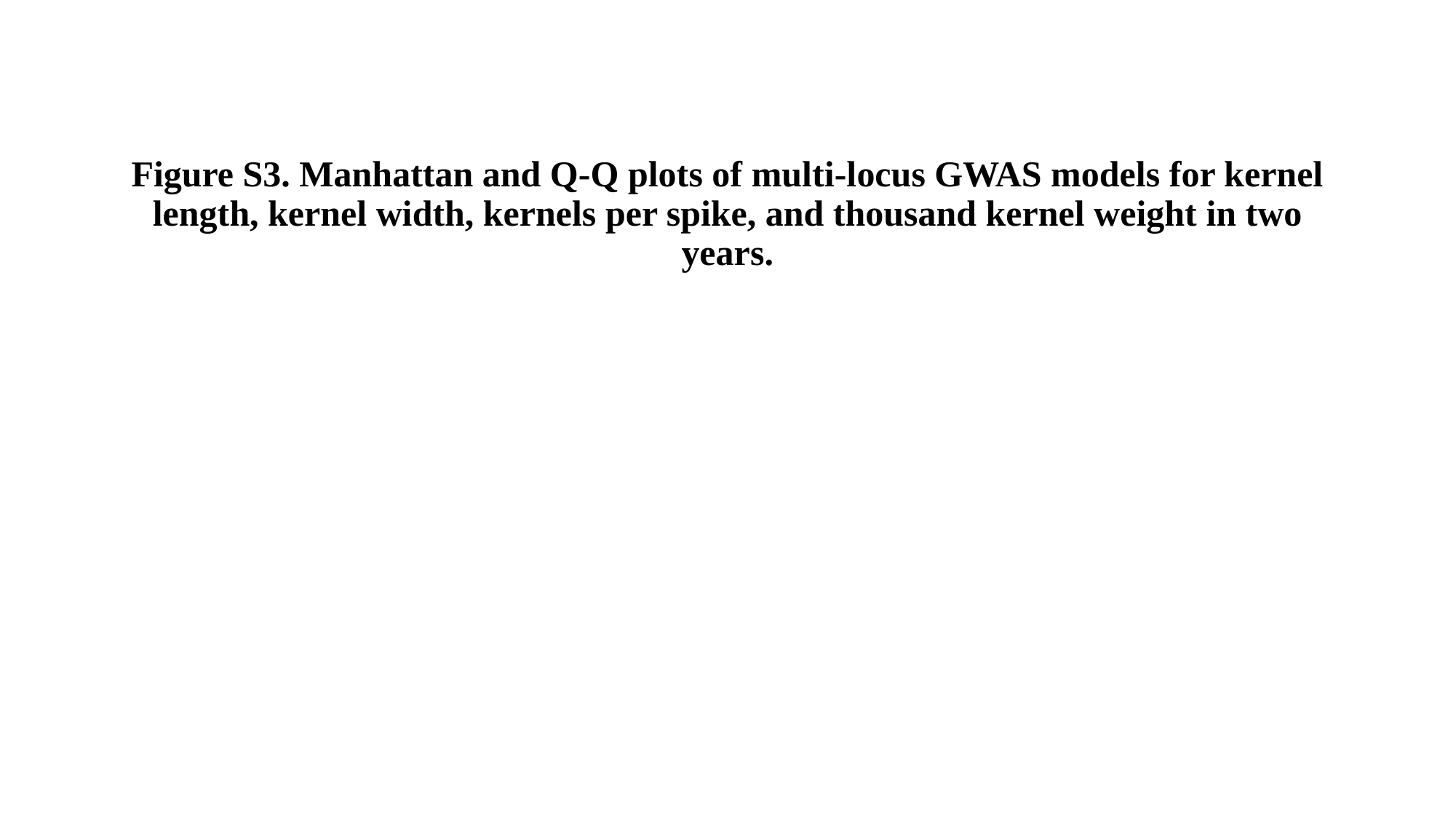

Figure S3. Manhattan and Q-Q plots of multi-locus GWAS models for kernel length, kernel width, kernels per spike, and thousand kernel weight in two years.

## Slide 6
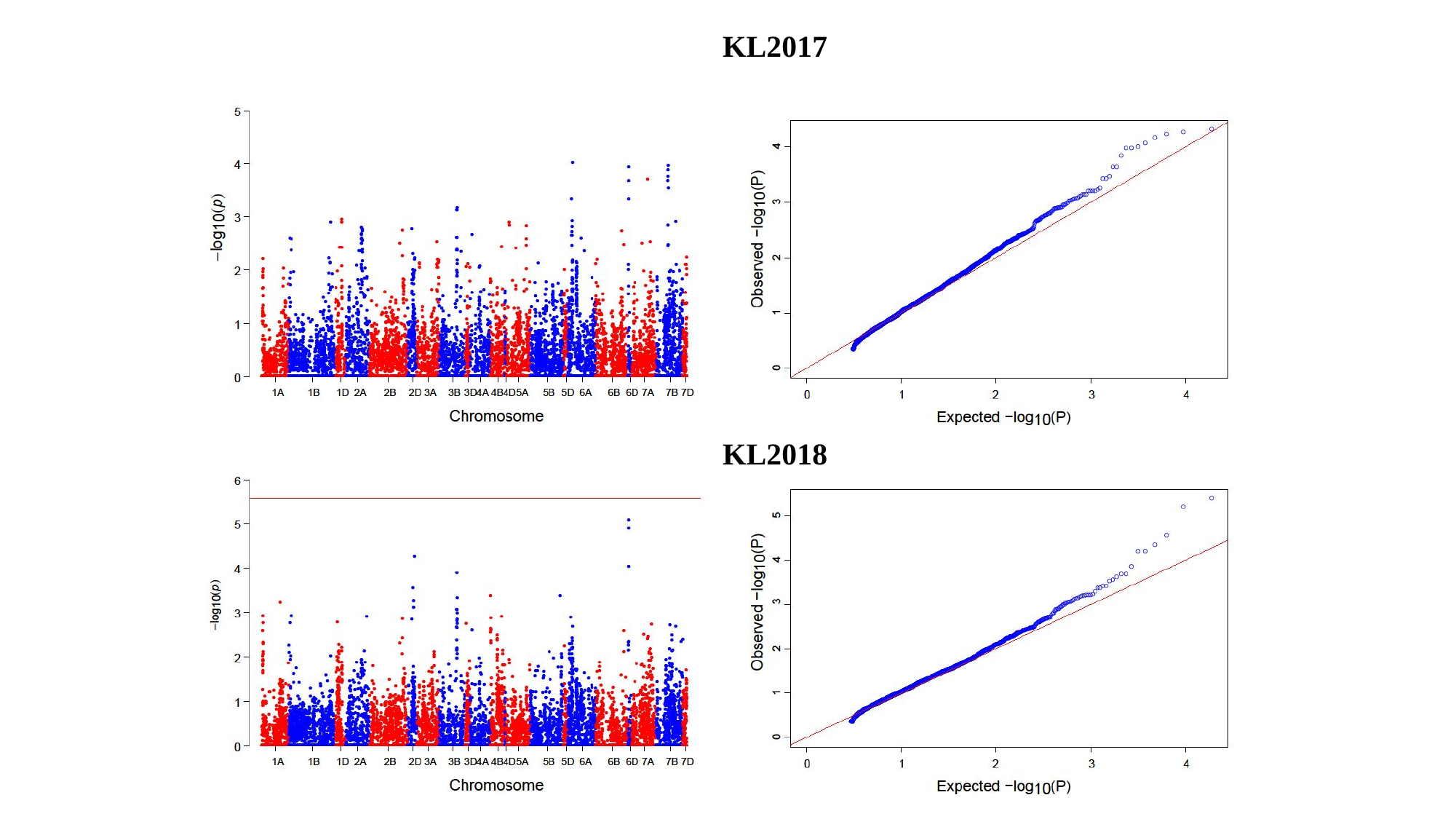

KL2017
KL2018

## Slide 7
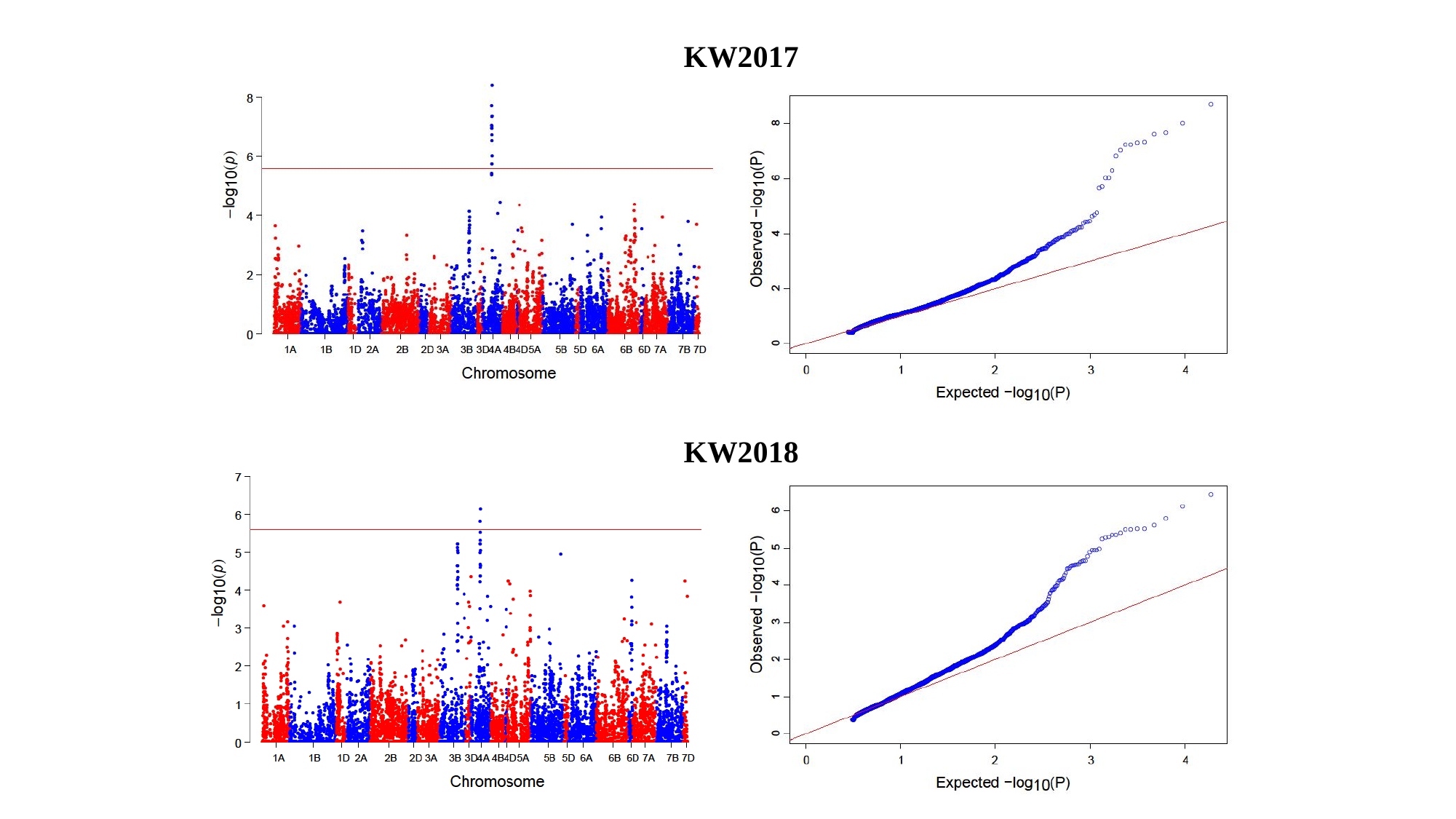

KW2017
KW2018

## Slide 8
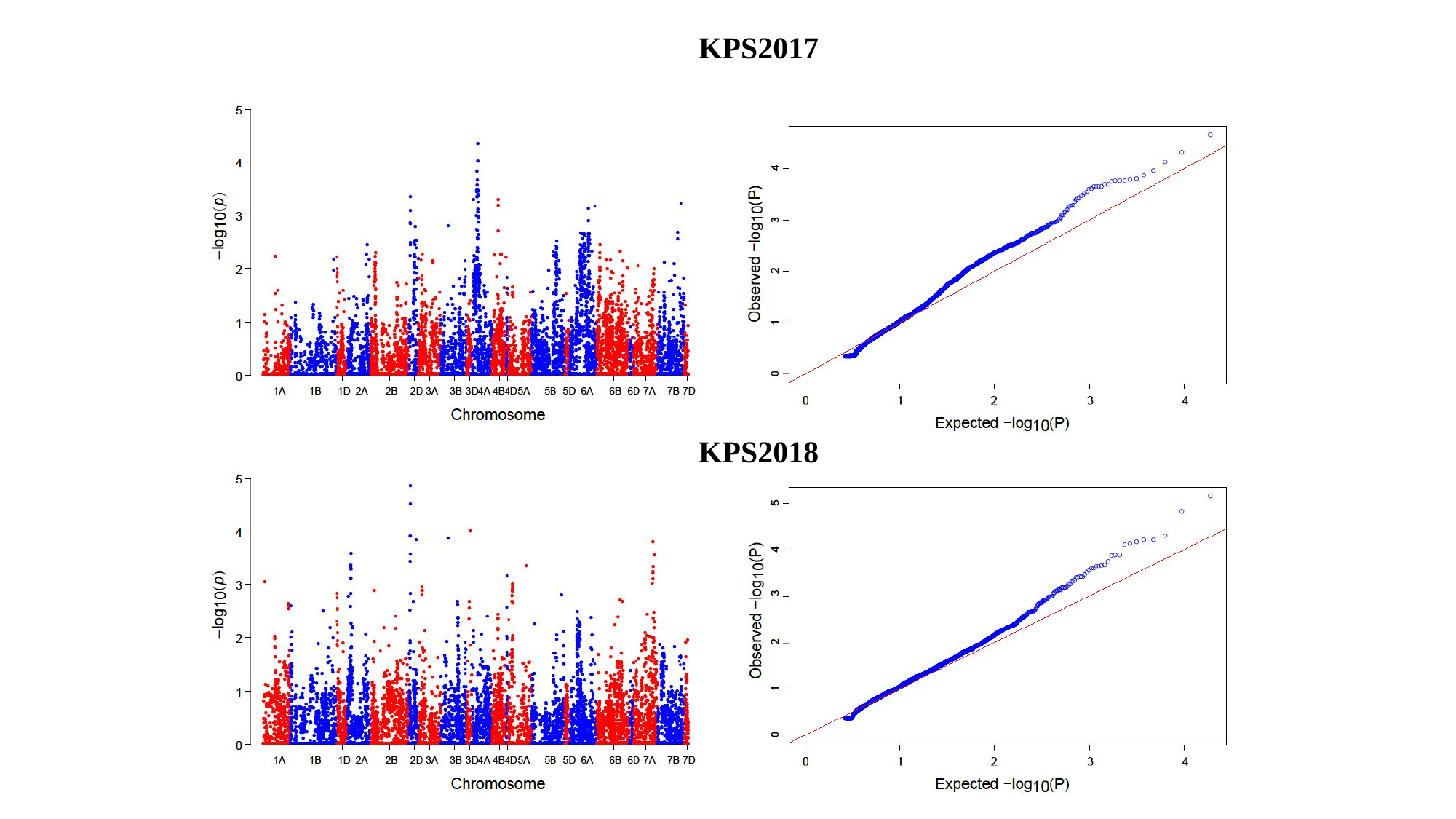

KPS2017
KPS2018

## Slide 9
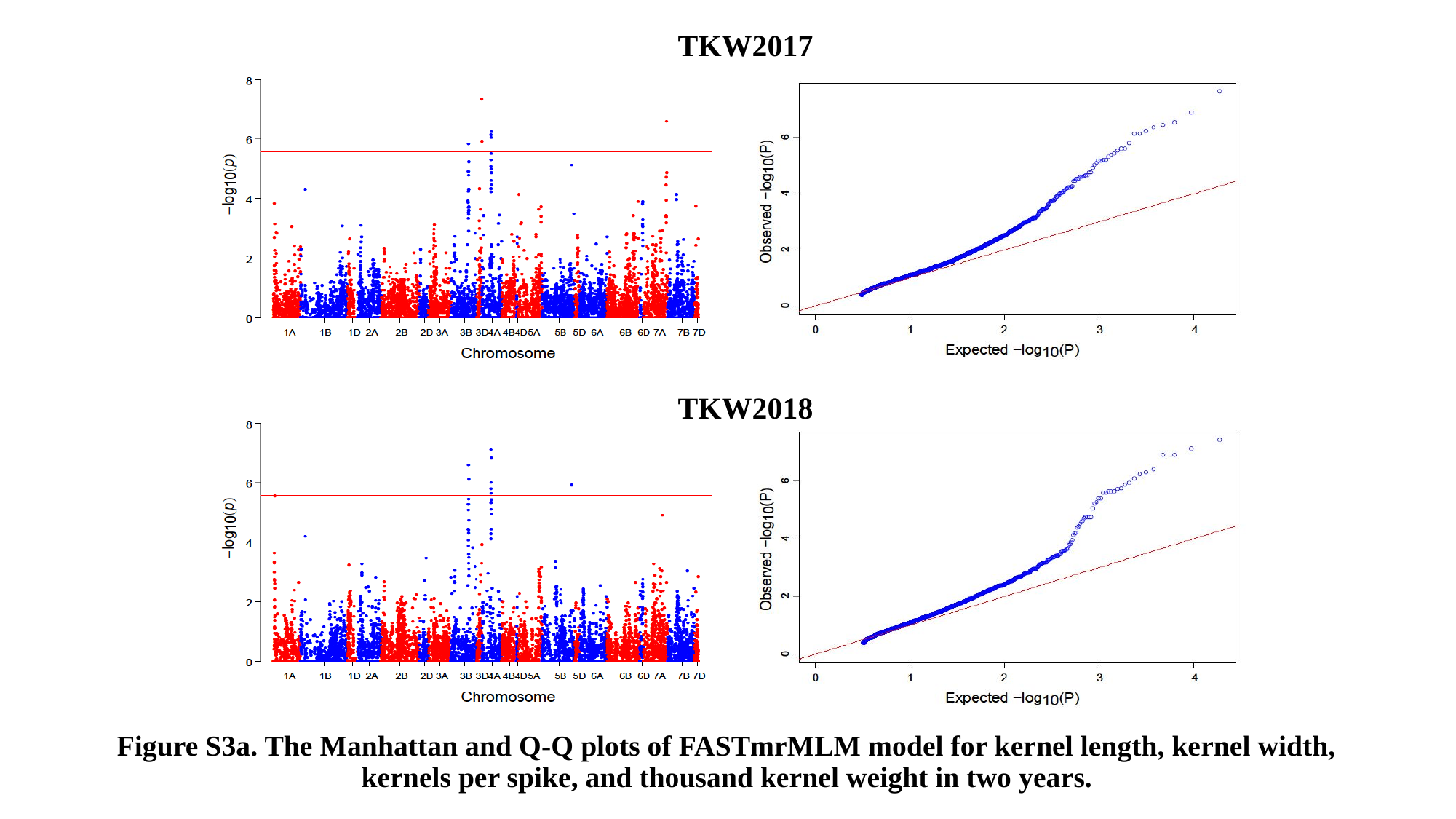

TKW2017
TKW2018
Figure S3a. The Manhattan and Q-Q plots of FASTmrMLM model for kernel length, kernel width, kernels per spike, and thousand kernel weight in two years.

## Slide 10
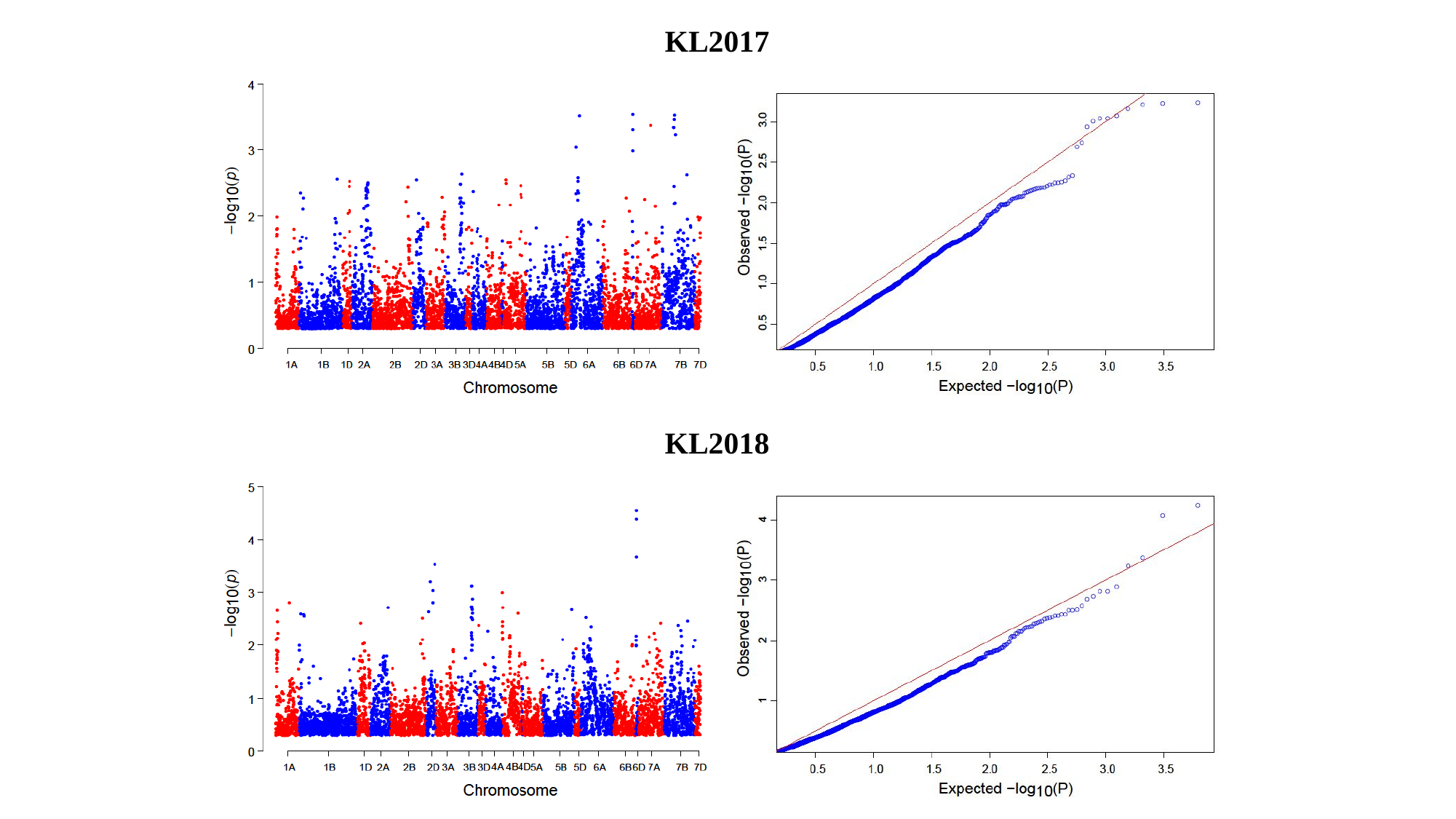

KL2017
KL2018

## Slide 11
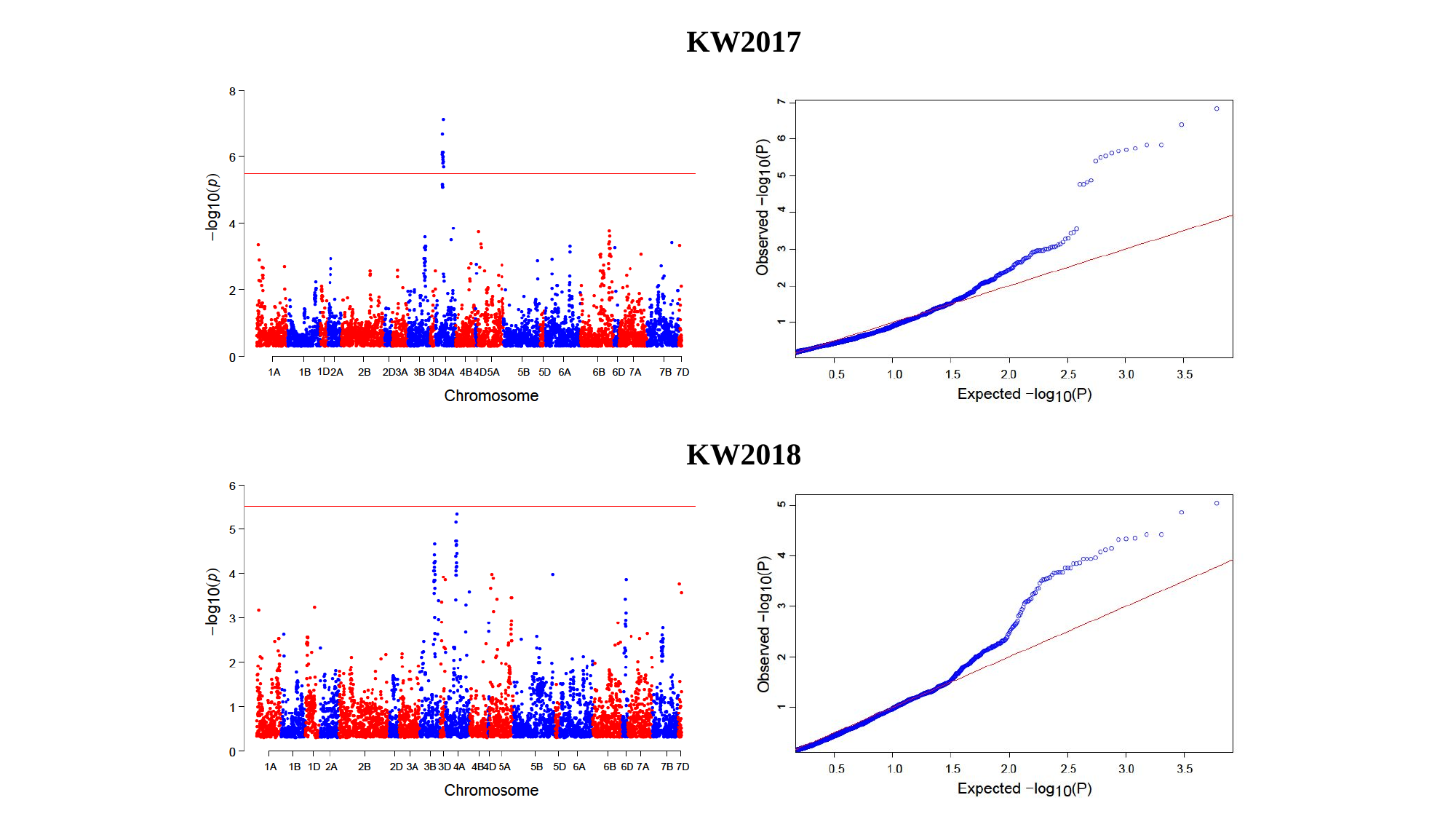

KW2017
KW2018

## Slide 12
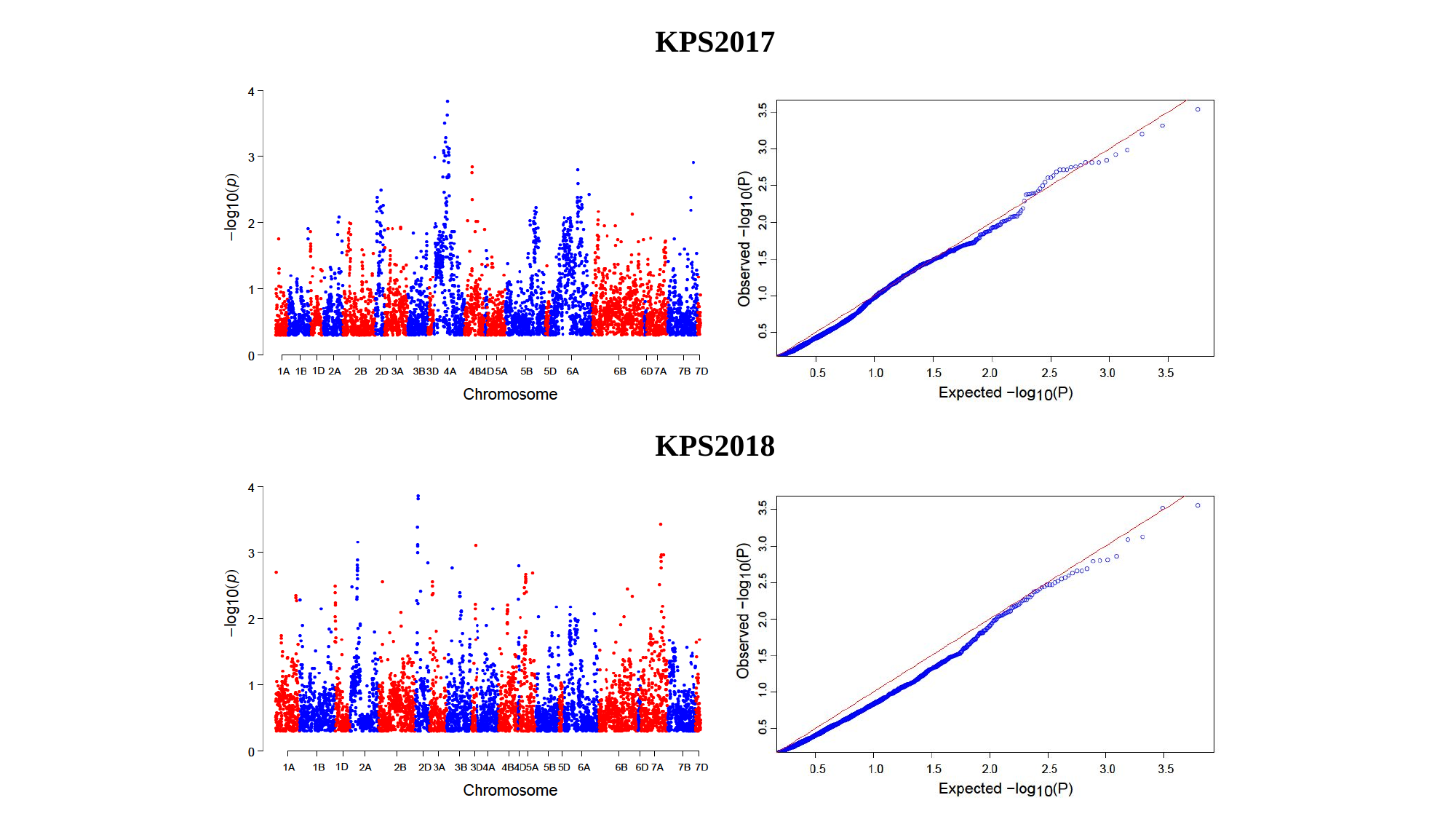

KPS2017
KPS2018

## Slide 13
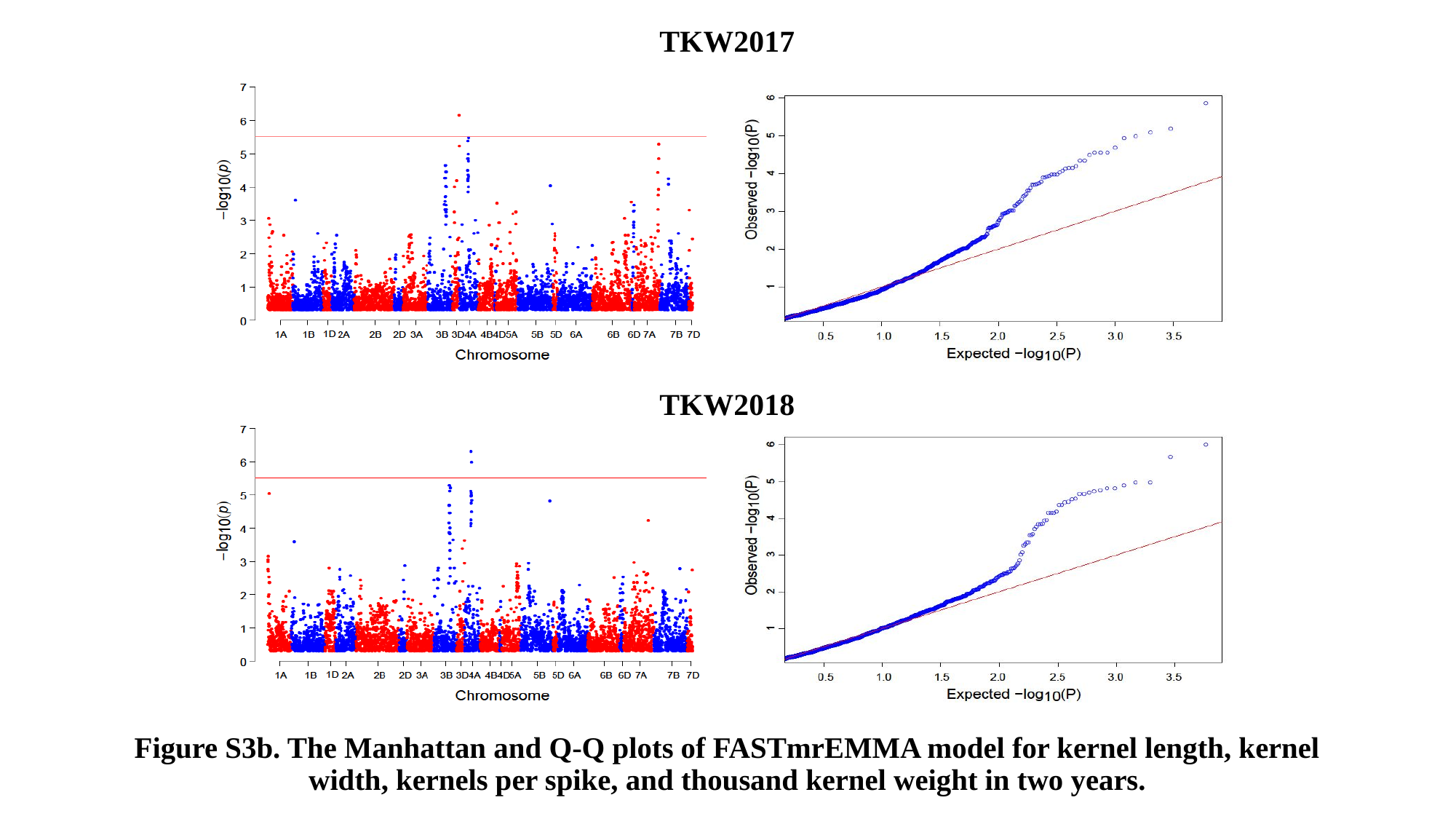

TKW2017
TKW2018
Figure S3b. The Manhattan and Q-Q plots of FASTmrEMMA model for kernel length, kernel width, kernels per spike, and thousand kernel weight in two years.

## Slide 14
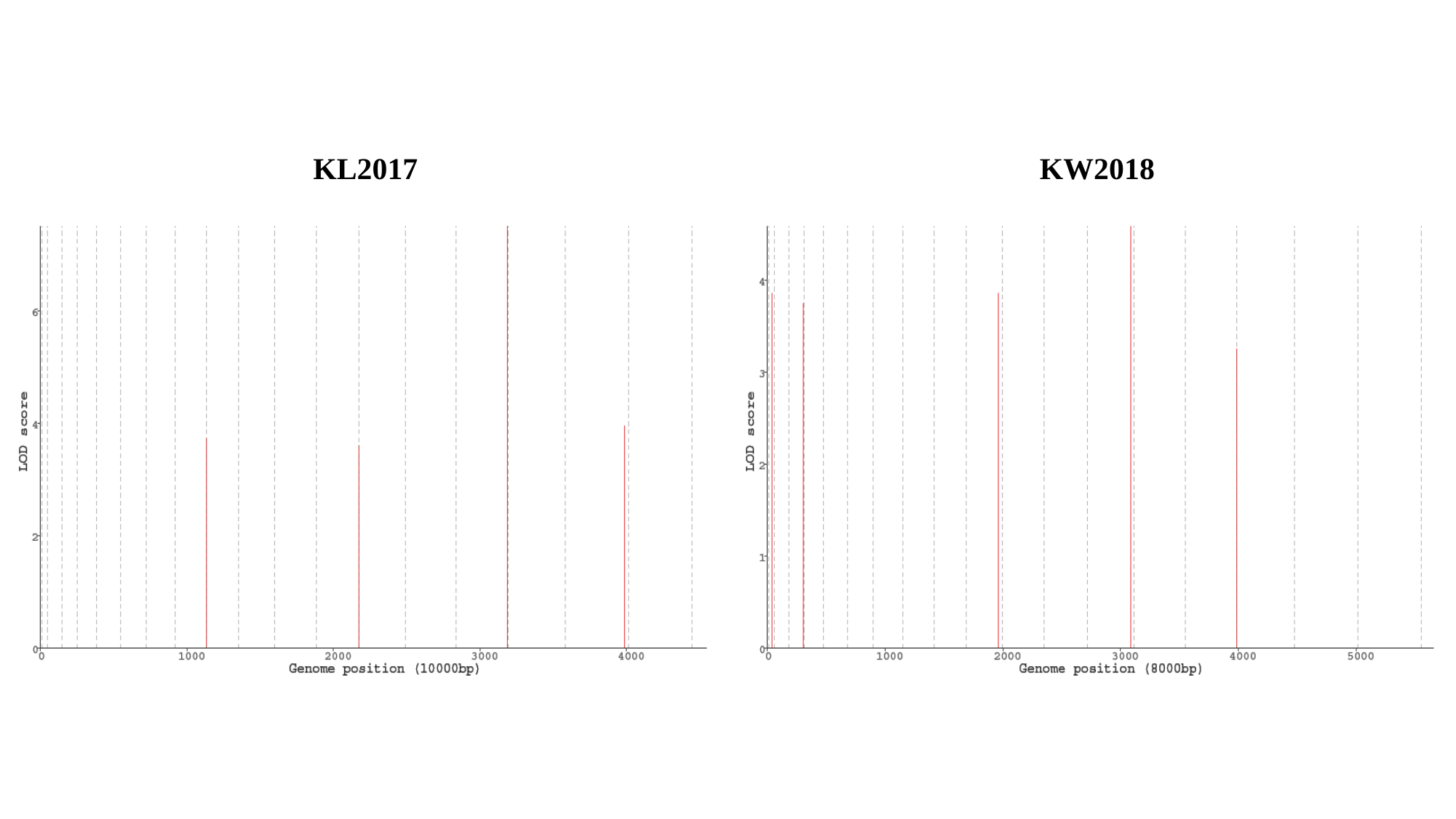

KL2017
KW2018

## Slide 15
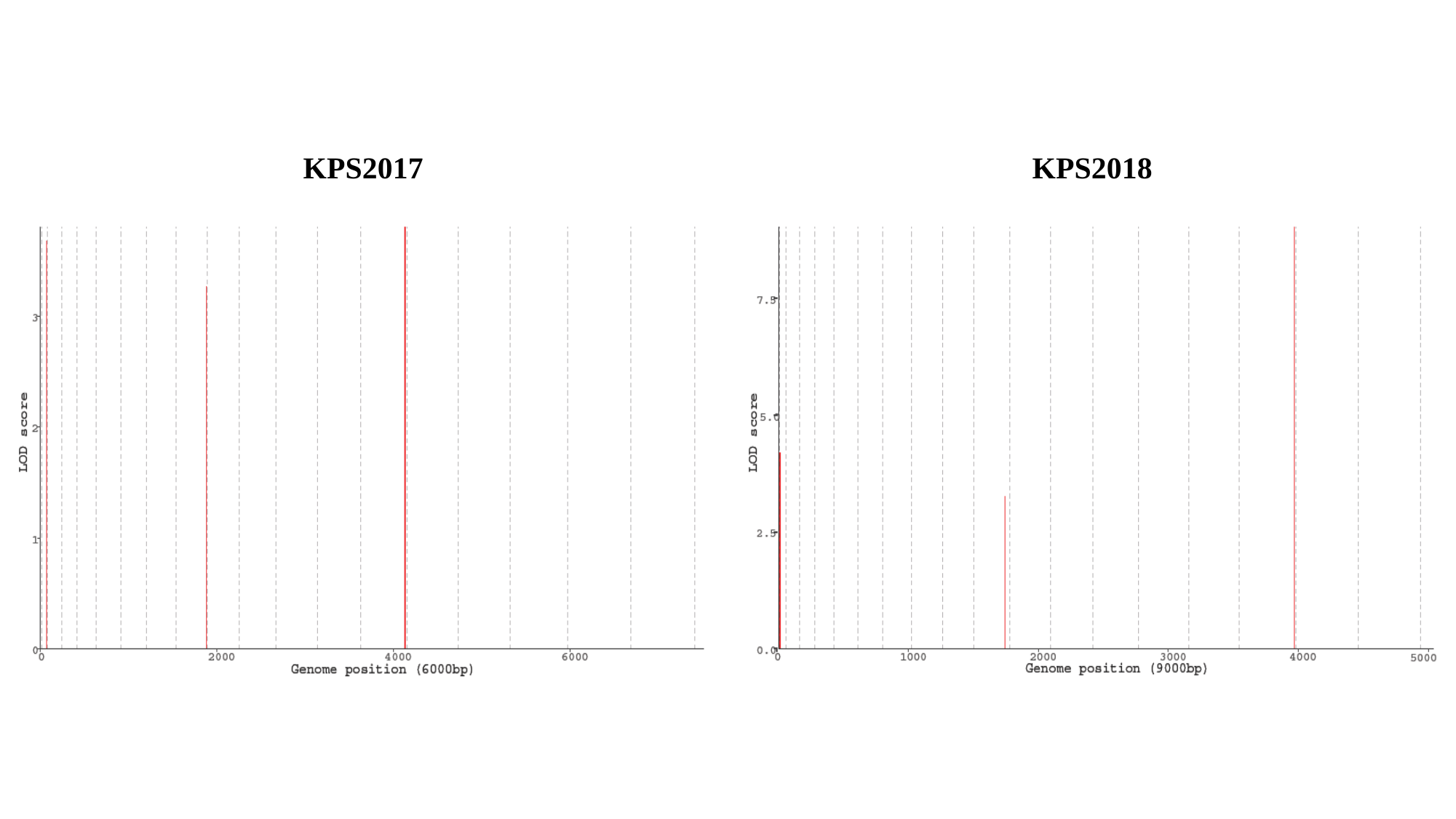

KPS2017
KPS2018

## Slide 16
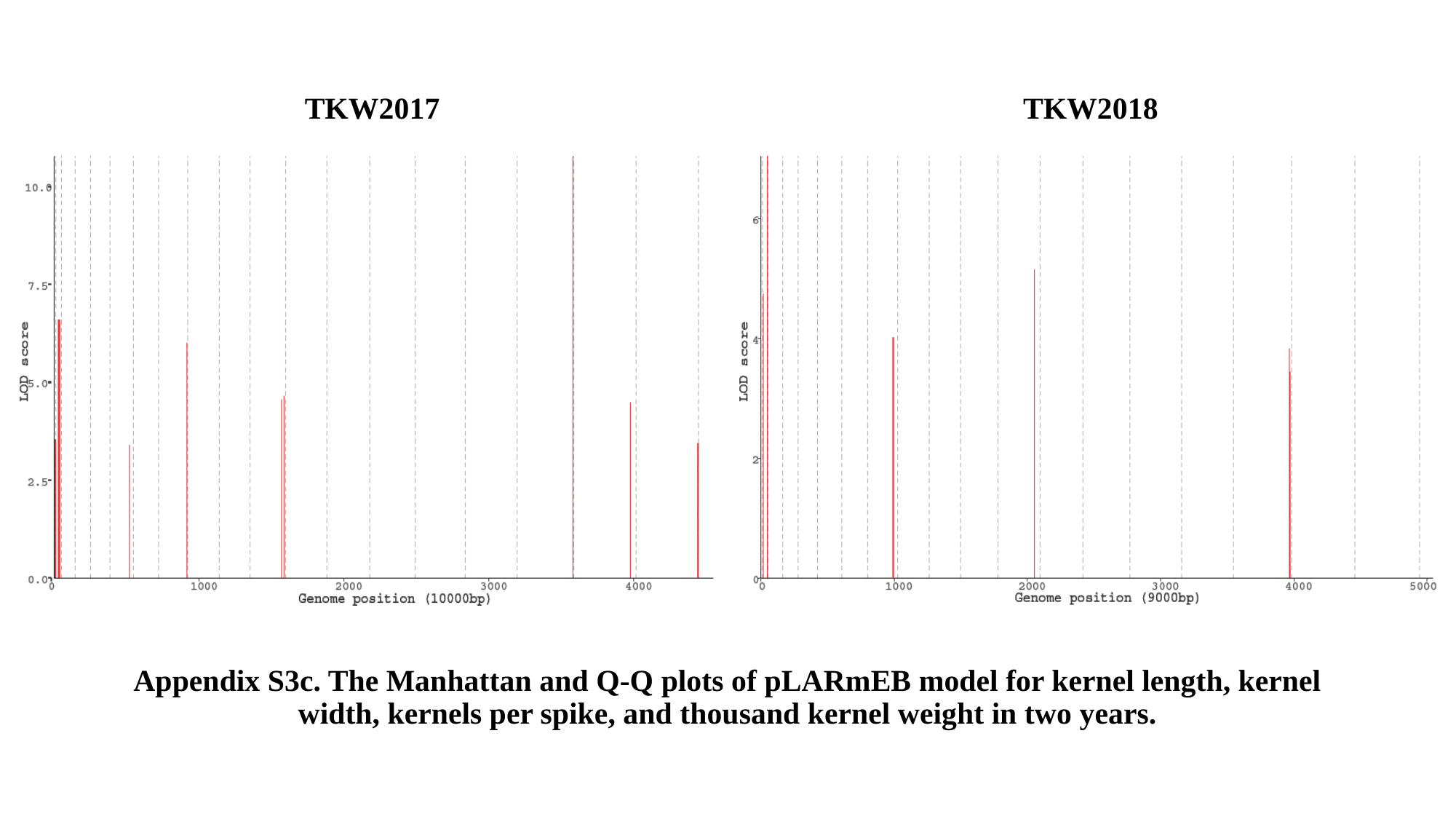

TKW2017
TKW2018
Appendix S3c. The Manhattan and Q-Q plots of pLARmEB model for kernel length, kernel width, kernels per spike, and thousand kernel weight in two years.

## Slide 17
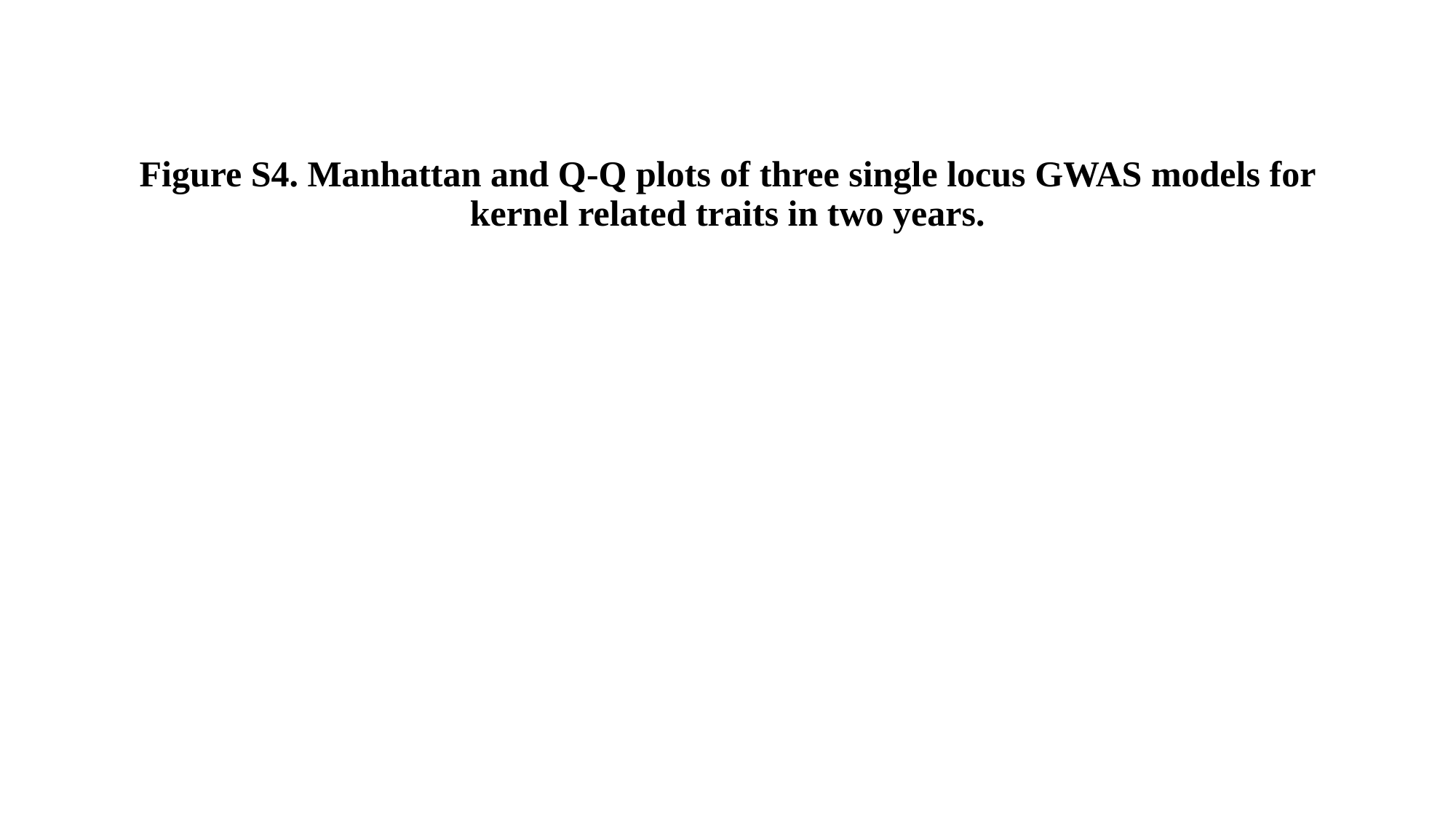

Figure S4. Manhattan and Q-Q plots of three single locus GWAS models for kernel related traits in two years.

## Slide 18
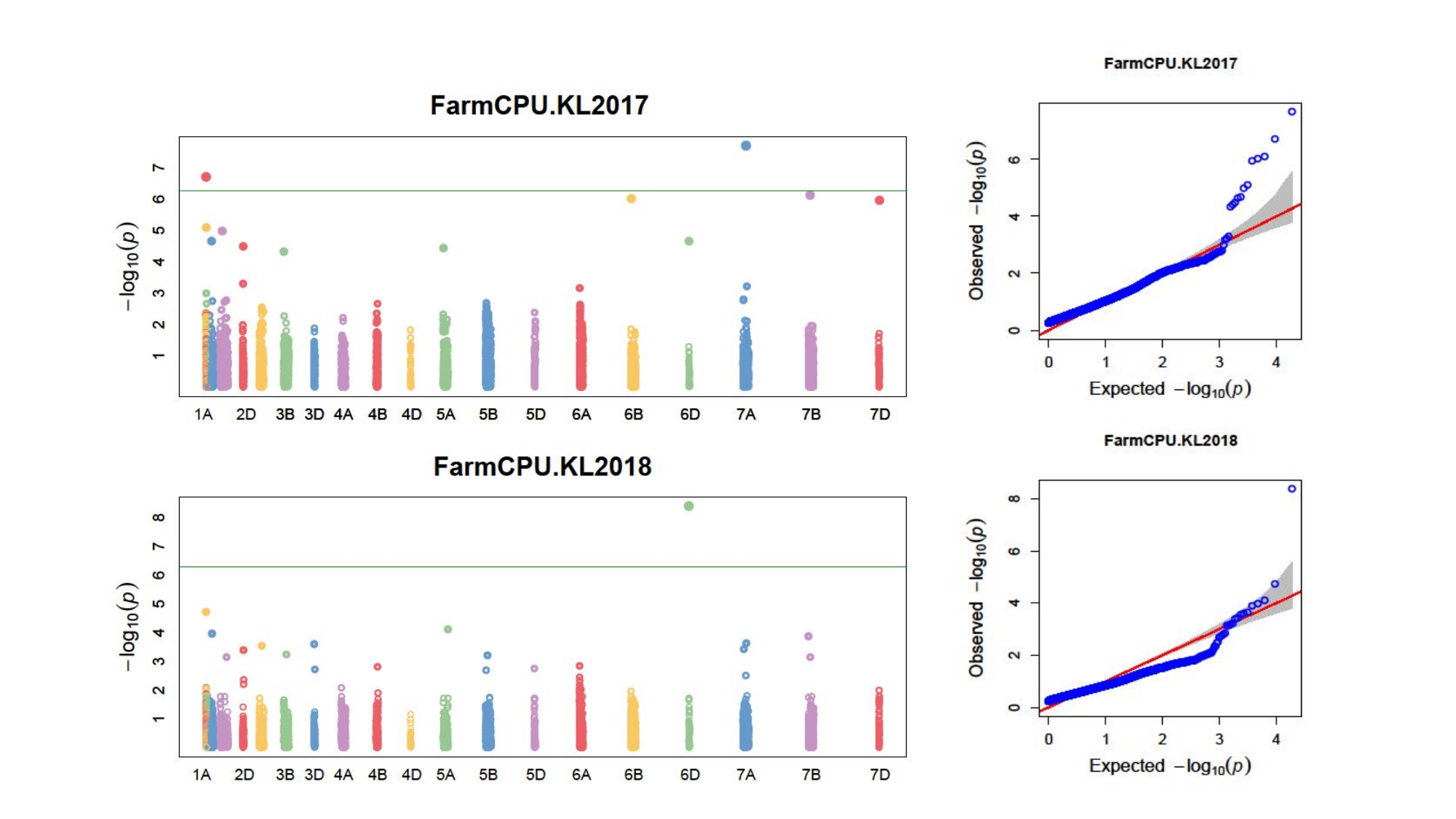

## Slide 19
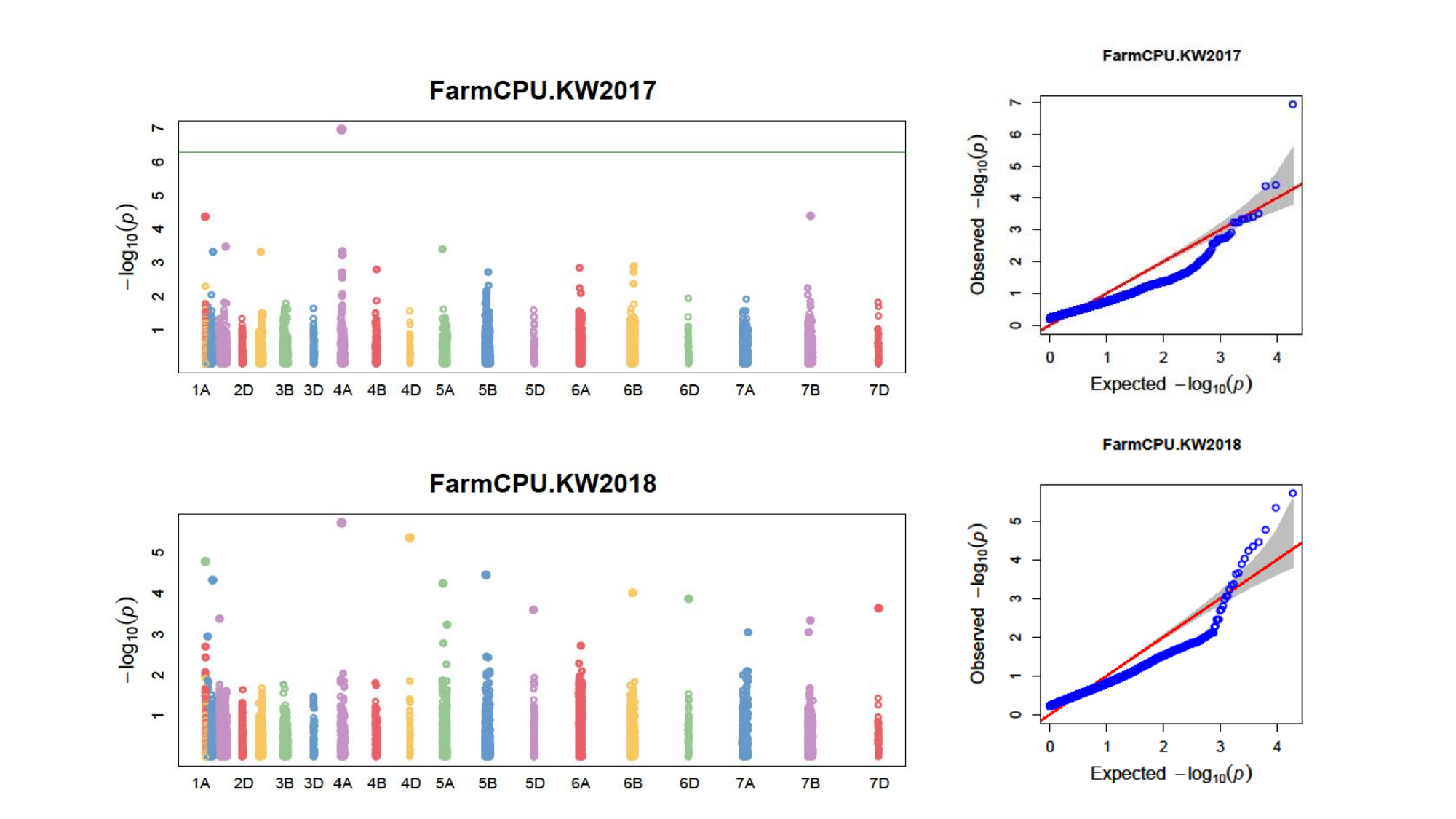

## Slide 20
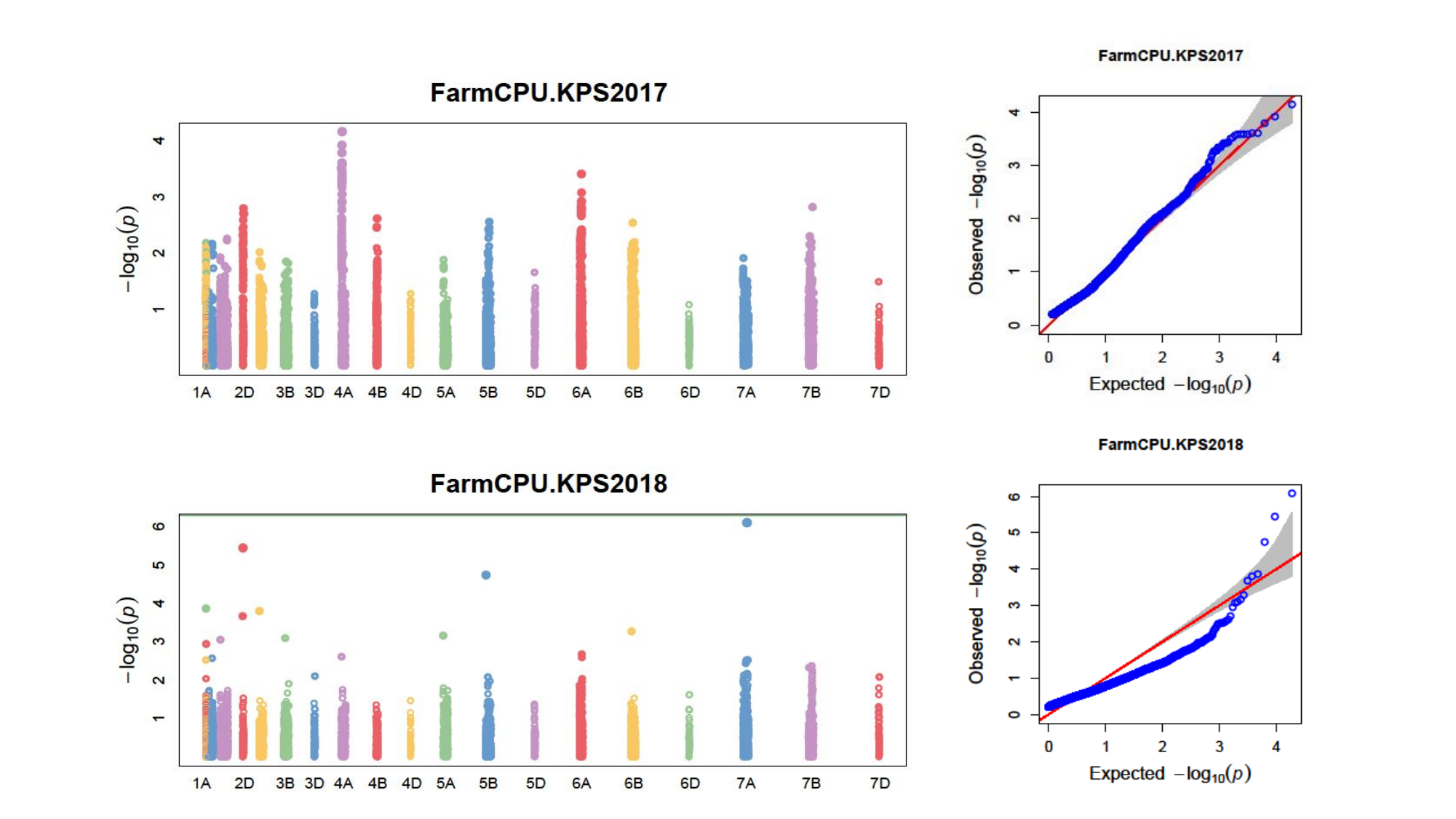

## Slide 21
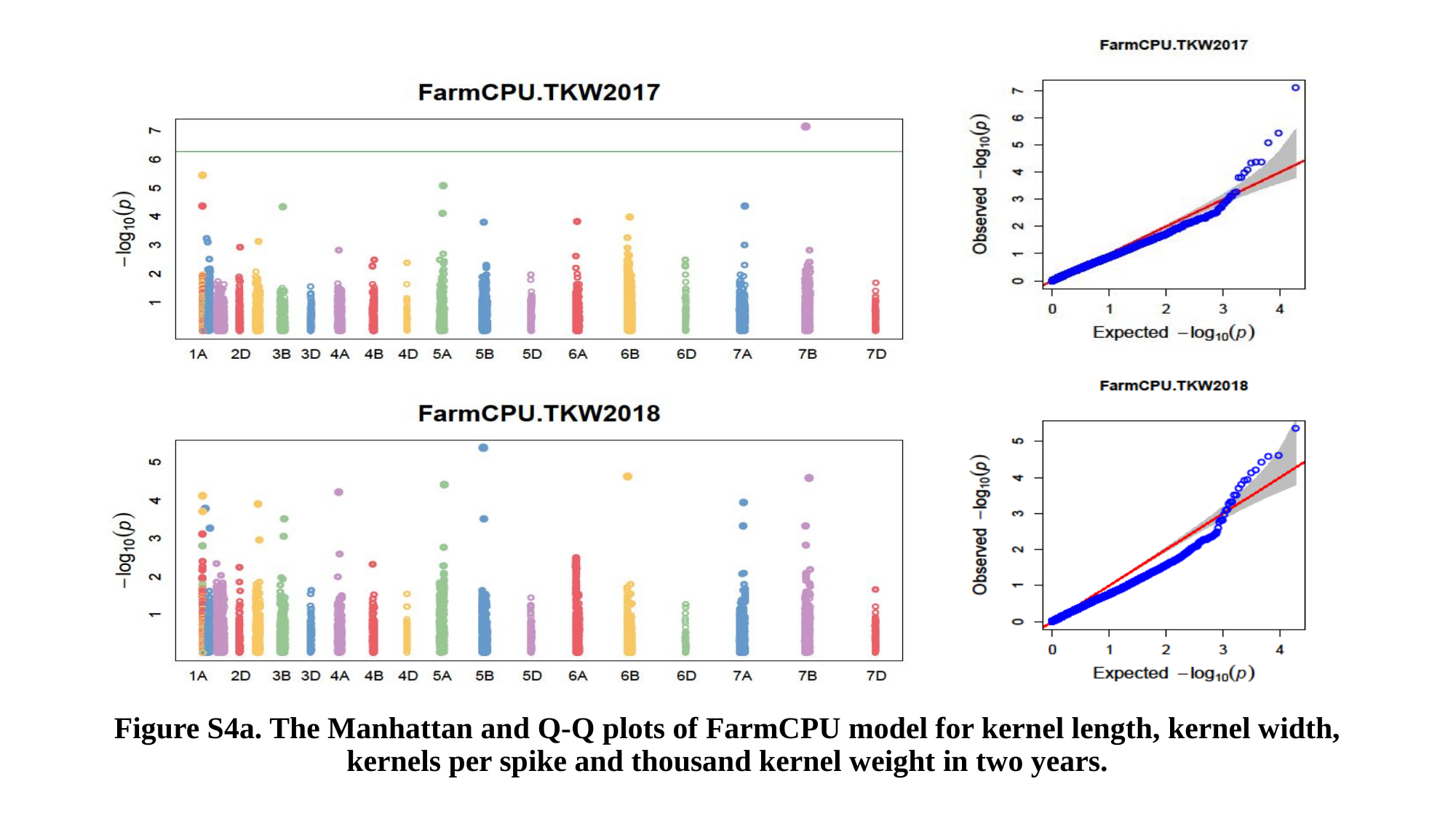

Figure S4a. The Manhattan and Q-Q plots of FarmCPU model for kernel length, kernel width, kernels per spike and thousand kernel weight in two years.

## Slide 22
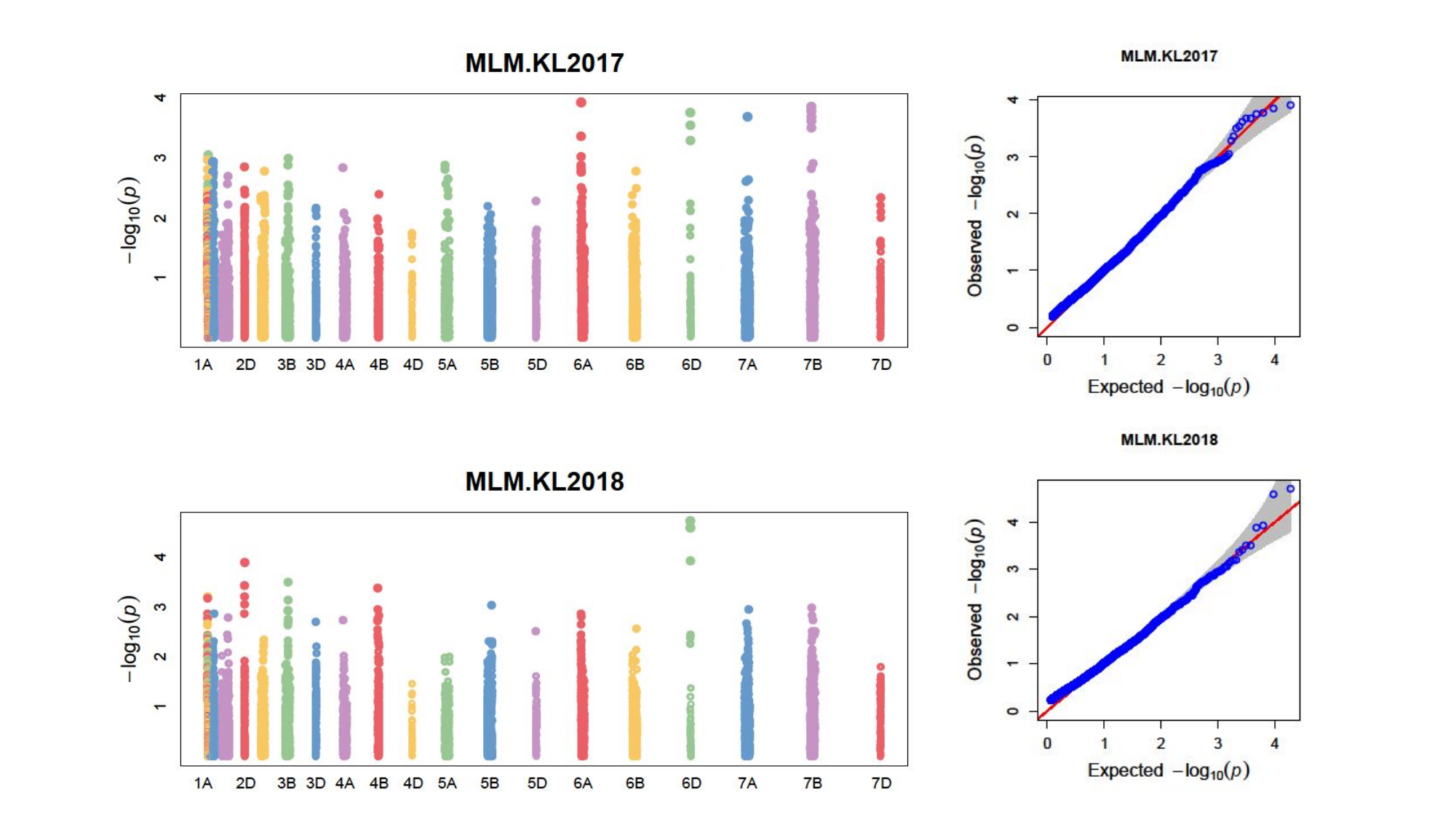

## Slide 23
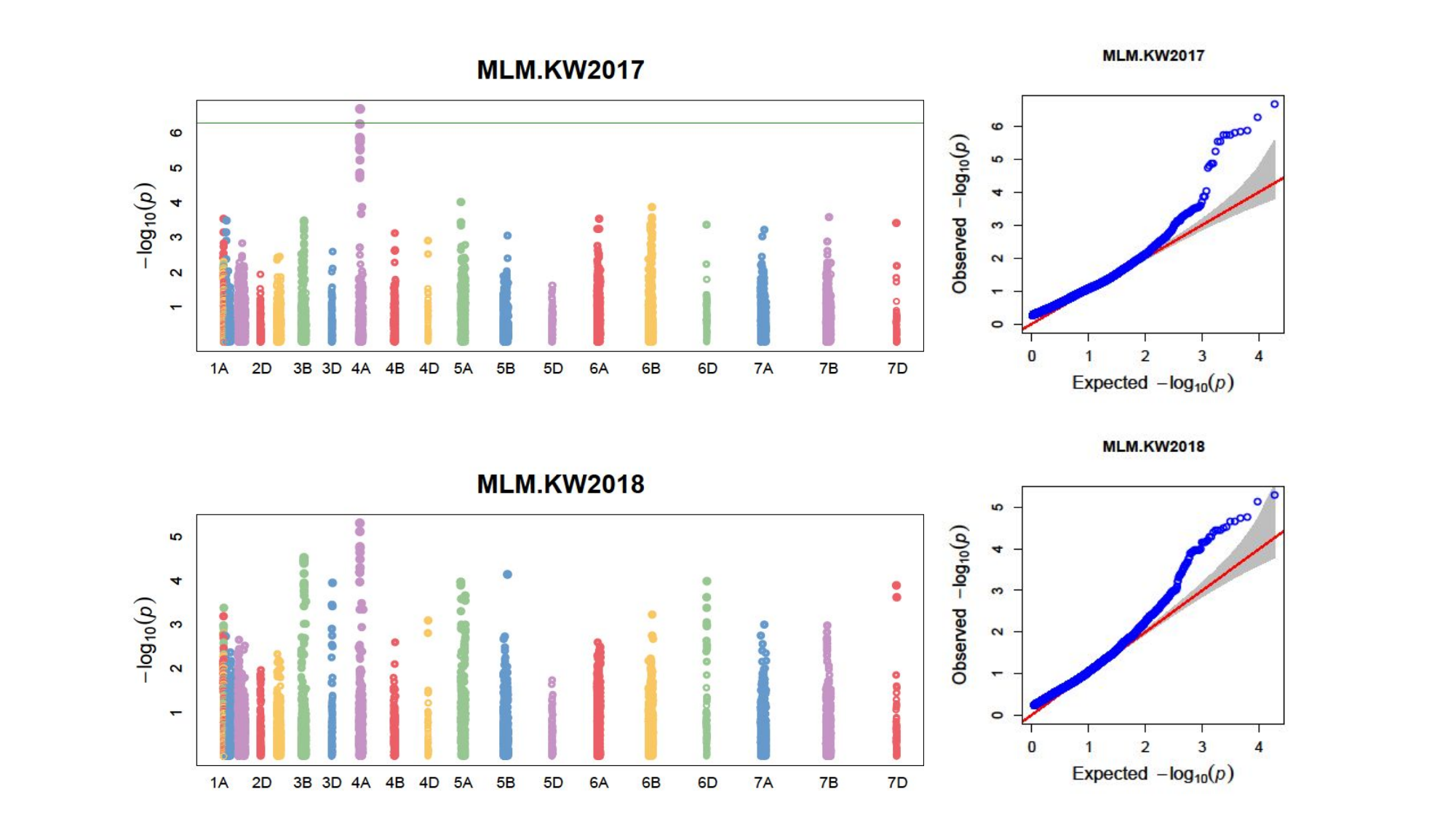

## Slide 24
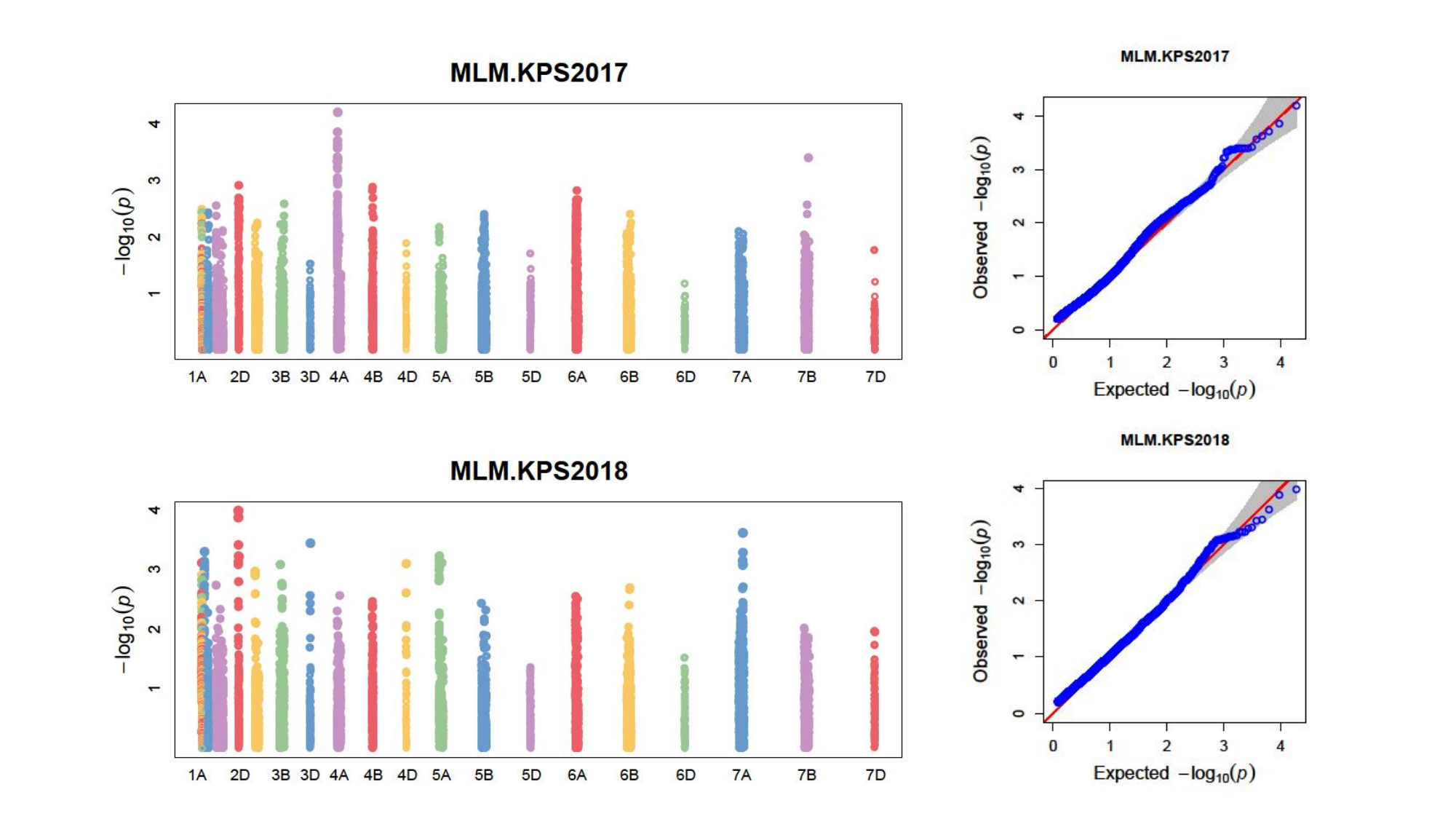

## Slide 25
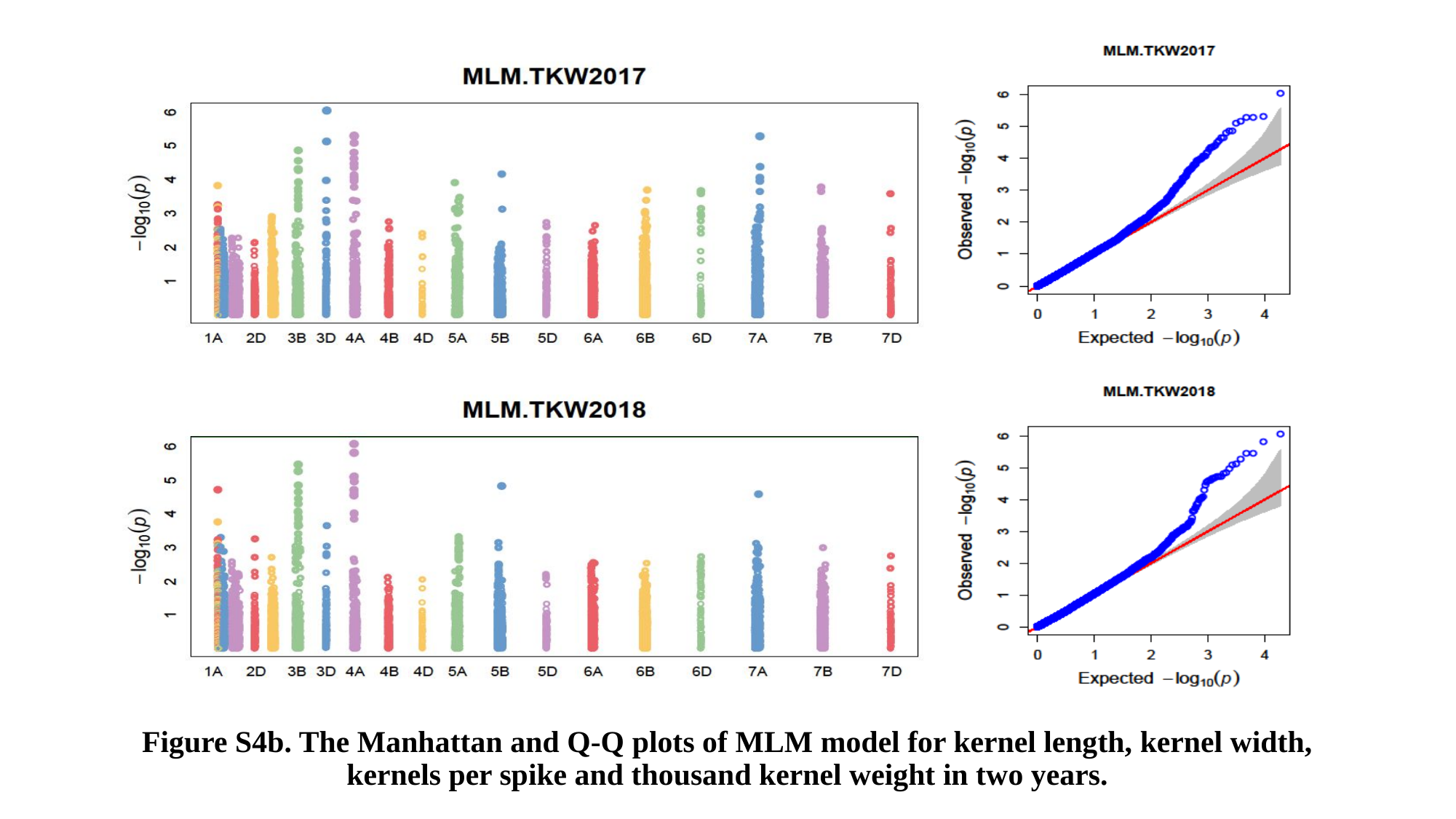

Figure S4b. The Manhattan and Q-Q plots of MLM model for kernel length, kernel width, kernels per spike and thousand kernel weight in two years.

## Slide 26
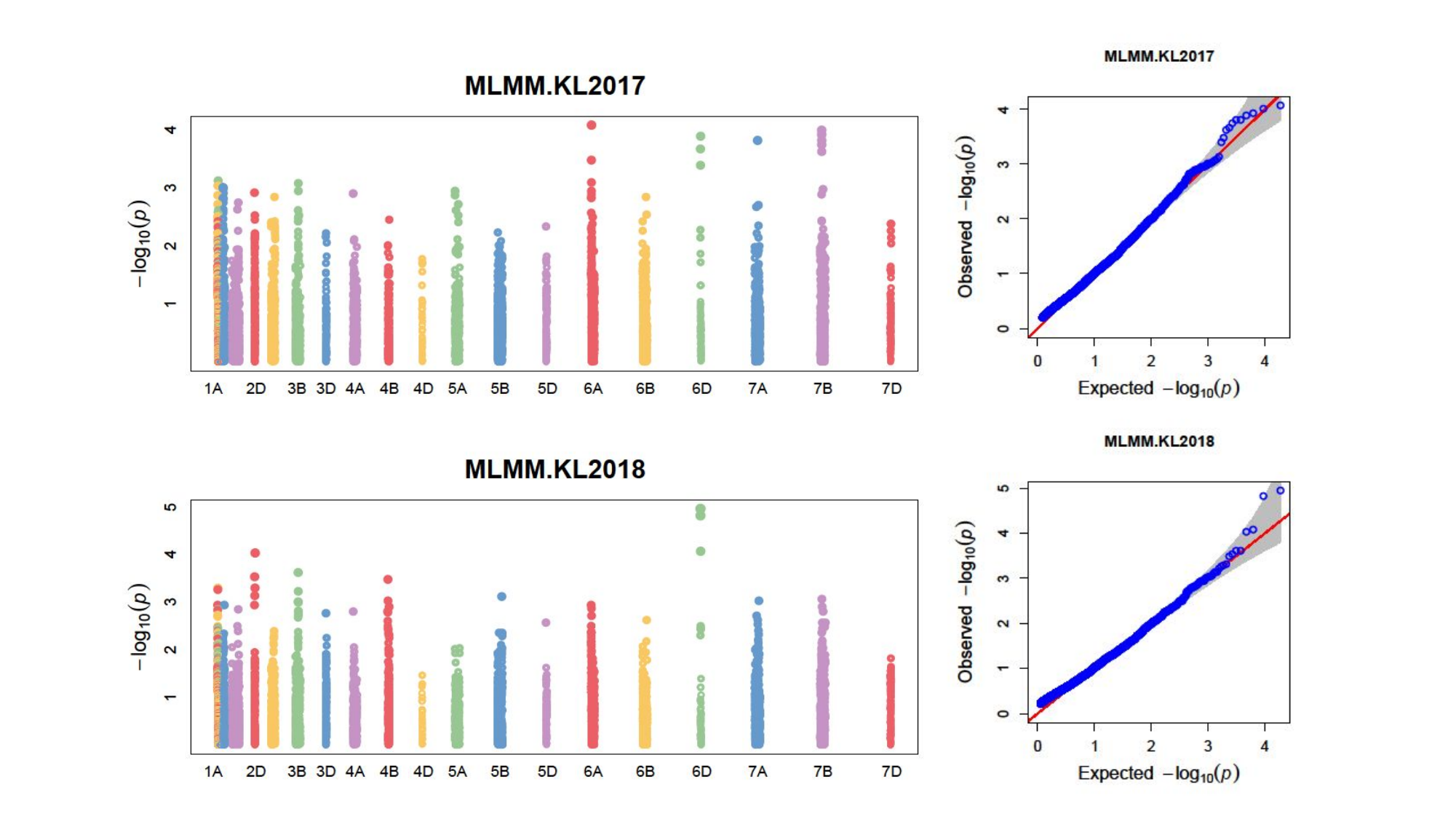

## Slide 27
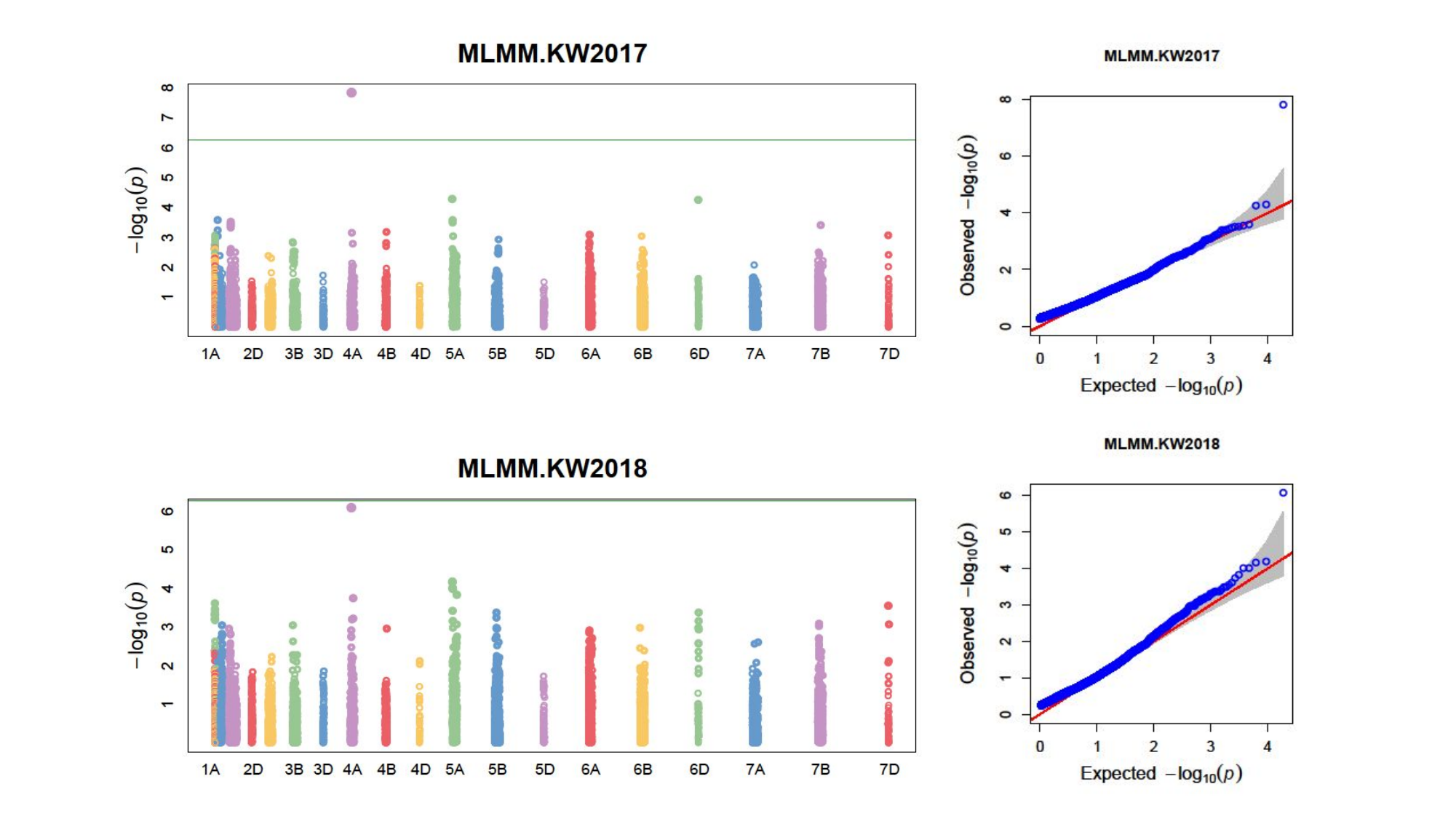

## Slide 28
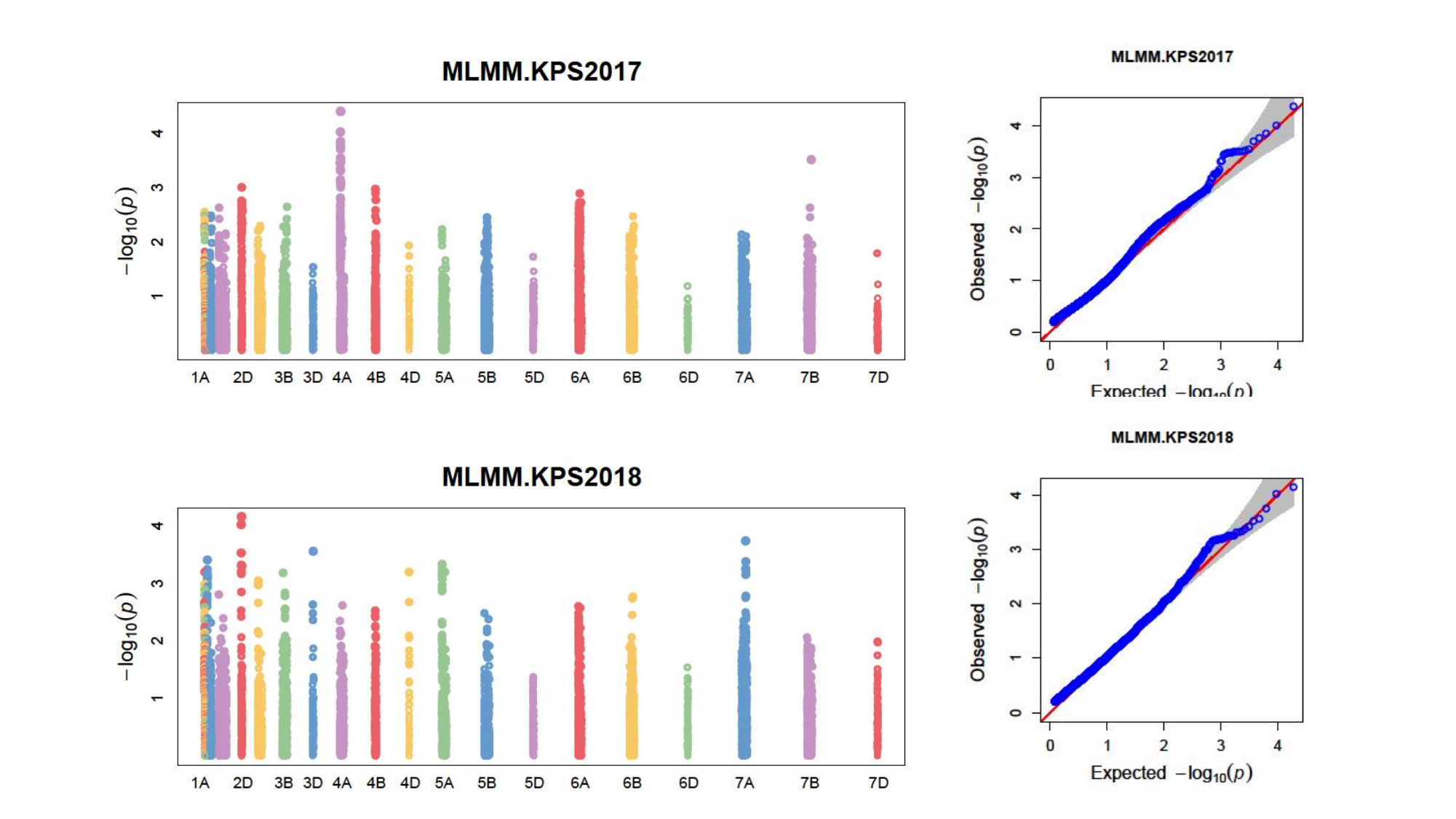

## Slide 29
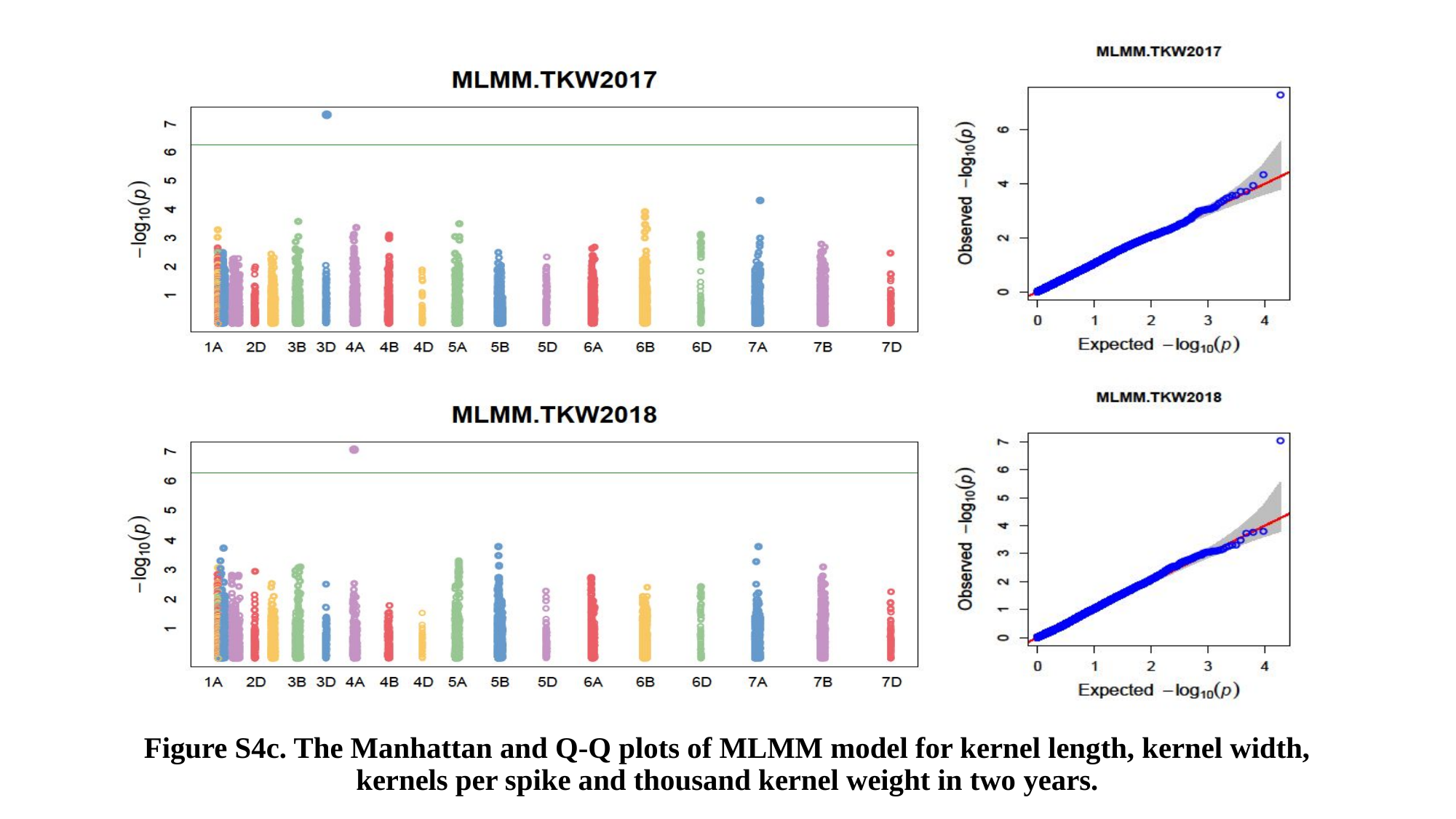

Figure S4c. The Manhattan and Q-Q plots of MLMM model for kernel length, kernel width, kernels per spike and thousand kernel weight in two years.
