# Supplementary material for: Appraising the Genetic Architecture of Kernel Traits in Hexaploid Wheat Using GWAS
Source: Int J Mol Sci. 2020 Aug 6;21(16):5649. doi: 10.3390/ijms21165649 (PMC7460857; doi:10.3390/ijms21165649)
Supplement: Supplementary file 1 [file ijms-21-05649-s001.zip › Supplementary data/Table S1-S6.docx]

**Table S1.** Phenotypic variation for kernel length, kernel width, kernels per spike, and thousand kernel weight in two years (2017-2018).

|  | **KL** | **KW** | **KPS** | **TKW** |
| --- | --- | --- | --- | --- |
| Mean | 7.53 | 3.82 | 46.73 | 43.59 |
| SD | 0.34 | 0.18 | 6.21 | 3.85 |
| C.V. | 4.53 | 4.58 | 13.28 | 8.84 |
| Min | 6.36 | 2.89 | 28.08 | 17.98 |
| Max | 8.62 | 4.37 | 68.93 | 56.99 |
| Skew | -0.26 | -0.75 | 0.16 | -1.05 |
| Kurtosis | 1.11 | 2.61 | 0.58 | 6.60 |

KL-Kernel length (mm); KW-Kernel width (mm); KPS-Kernels per spike; TKW-Thousand kernel weight (g).

**Table S2.** Analysis of Variance (ANOVA) and heritability for kernel length, kernel width, kernels per spike, and thousand kernel weight.

| **Trait** | **Source** | **SS** | **DF** | **MS** | **F-calculated** | **P-value** | **F-critical** | ***H^2^*** |
| --- | --- | --- | --- | --- | --- | --- | --- | --- |
| KL | Genotype | 221.8 | 317 | 0.70 | 18.83 | 0.00 | 1.15 | 0.89 |
|  | Year | 2.0 | 1 | 1.95 | 52.61 | 0.00 | 3.85 |  |
|  | Geno × Year | 25.3 | 317 | 0.08 | 2.15 | 0.00 | 1.15 |  |
|  | Error | 47.3 | 1272 | 0.04 |  |  |  |  |
| KW | Genotype | 58.2 | 317 | 0.18 | 9.67 | 0.00 | 1.15 | 0.84 |
|  | Year | 13.0 | 1 | 12.96 | 681.84 | 0.00 | 3.85 |  |
|  | Geno × Year | 9.4 | 317 | 0.03 | 1.55 | 0.00 | 1.15 |  |
|  | Error | 24.2 | 1272 | 0.02 |  |  |  |  |
| KPS | Genotype | 73240.1 | 317 | 231.04 | 11.08 | 0.00 | 1.15 | 0.80 |
|  | Year | 5399.1 | 1 | 5399.06 | 258.98 | 0.00 | 3.85 |  |
|  | Geno × Year | 15732.1 | 317 | 49.63 | 2.38 | 0.00 | 1.15 |  |
|  | Error | 26518.3 | 1272 | 20.85 |  |  |  |  |
| TKW | Genotype | 28234.2 | 317 | 89.07 | 9.80 | 0.00 | 1.15 | 0.85 |
|  | Year | 468.2 | 1 | 468.18 | 51.51 | 0.00 | 3.85 |  |
|  | Geno × Year | 4299.8 | 317 | 13.56 | 1.49 | 0.00 | 1.15 |  |
|  | Error | 11560.4 | 1272 | 9.09 |  |  |  |  |

DF (Degree of freedom); SS (Sum of Square); MS (Mean square); *H^2^* (Heritability); KL (Kernel length); KW (Kernel width); KPS (Kernels per spike); TKW (Thousand kernel weight).

Table S3. Significant SNPs detected by three single-locus GWAS methods in two years for kernel length, kernel width, kernels per spike, and thousand kernel weight.

| **Trait** | **Year** | **Method** | **SNPs** | **Chr** | **Position (bp)** | ***P*-Value** |
| --- | --- | --- | --- | --- | --- | --- |
| KL | 2017 | FarmCPU | BS00009104_51 | 1A | 325 | 1.94E-07 |
| KL | 2017 | FarmCPU | BobWhite_09733_301 | 1B | 868 | 8.24E-06 |
| KL | 2017 | FarmCPU | wsnp_Ex_28204_37349164 | 2A | 285,421 | 2.29E-05 |
| KL | 2017 | FarmCPU | Excalibur_09344_137 | 2B | 559,774 | 1.05E-05 |
| KL | 2017 | FarmCPU | Kukri_2561_179 | 2D | 985,251 | 3.21E-05 |
| KL | 2017 | FarmCPU | BS00003696_51 | 5A | 2,163,100 | 3.78E-05 |
| KL | 2017 | FarmCPU | RAC875_c76124_264 | 6B | 3,171,269 | 9.61E-07 |
| KL | 2017 | FarmCPU | Kukri_c8827_217 | 7A | 3,732,575 | 2.04E-08 |
| KL | 2017 | FarmCPU | Tdurum_contig102328_129 | 7B | 4,030,937 | 7.8E-07 |
| KL | 2017 | FarmCPU | D_contig65328_393 | 7D | 4,327,151 | 1.13E-06 |
| KL | 2018 | FarmCPU | BS00011695_51 | 1B | 628 | 1.91E-05 |
| KL | 2018 | MLMM | Kukri_07961_350 | 6D | 3,491,830 | 1.54E-05 |
| KL | 2018 | MLMM | Kukri_07961_503 | 6D | 3,491,885 | 1.11E-05 |
| KW | 2017 | FarmCPU | Excalibur_rep_001018_254 | 1A | 426 | 4.19E-05 |
| KW | 2017 | MLM | D_GCE8AKX01AOOSX_177 | 4A | 1,800,619 | 1.91E-05 |
| KW | 2017 | MLM | GENE_2778_24 | 4A | 1,801,076 | 1.64E-05 |
| KW | 2017 | MLM | IAAV4932 | 4A | 1,801,532 | 1.38E-05 |
| KW | 2017 | MLM | IAAV708 | 4A | 1,801,761 | 1.38E-05 |
| KW | 2017 | MLM | Kukri_006747_240 | 4A | 1,801,989 | 2.85E-06 |
| KW | 2017 | MLM | wsnp_Ex_07338_26018247 | 4A | 1,802,674 | 3.04E-06 |
| KW | 2017 | MLM | wsnp_Ra_0022_2067517 | 4A | 1,804,272 | 1.84E-06 |
| KW | 2017 | MLM | Kukri_rep_005552_396 | 4A | 1,804,730 | 6.06E-06 |
| KW | 2017 | FarmCPU | Tdurum_contig15734_221 | 7B | 4,127,623 | 3.95E-05 |
| KW | 2018 | FarmCPU | CAP7_c973_156 | 1D | 2,194 | 1.70E-05 |
| KW | 2018 | FarmCPU | wsnp_BE444858D_Ta_1_1 | 4D | 2,144,451 | 4.52E-06 |
| KW | 2018 | FarmCPU | RAC875_c99618_116 | 5B | 2,553,545 | 3.55E-05 |
|  |  |  |  |  |  | **(Continued)** |
| **Table S3** | |  |  |  |  | **Continued** |
| **Trait** | **Year** | **Method** | **SNPs** | **Chr** | **Position (bp)** | ***P*-Value** |
| KW | 2018 | MLM | BobWhite_rep_c67207_126 | 3B | 1,501,266 | 3.50E-05 |
| KW | 2018 | MLM | IACX8336 | 3B | 1,502,502 | 3.46E-05 |
| KW | 2018 | MLM | RAC875_0057_224 | 3B | 1,502,810 | 3.50E-05 |
| KW | 2018 | MLM | wsnp_Ex_07303_25979191 | 3B | 1,504,664 | 2.92E-05 |
| KW | 2018 | MLM | wsnp_Ku_03721_21798677 | 3B | 1,508,371 | 3.93E-05 |
| KW | 2018 | MLM | BS00079388_51 | 4A | 1,800,162 | 3.18E-05 |
| KW | 2018 | MLM | BS00079389_51 | 4A | 1,800,391 | 2.25E-05 |
| KW | 2018 | MLM | Ex_07338_401 | 4A | 1,800,847 | 7.45E-06 |
| KW | 2018 | MLM | Kukri_c67793_237 | 4A | 1,802,217 | 1.70E-05 |
| KW | 2018 | MLM | wsnp_Ex_21165_30292808 | 4A | 1,802,902 | 2.25E-05 |
| KW | 2018 | MLM | wsnp_Ex_c4068_7351806 | 4A | 1,803,131 | 1.84E-05 |
| KW | 2018 | MLMM | BS00021738_51 | 4A | 1,804,501 | 8.18E-07 |
| KPS | 2017 | MLMM | wsnp_Ra_rep_007017_90667618 | 4A | 1,774,526 | 4.07E-05 |
| KPS | 2018 | FarmCPU | BS00000905_51 | 2D | 974,024 | 3.71E-06 |
| KPS | 2018 | FarmCPU | Excalibur_c45323_708 | 5B | 2,484,878 | 1.79E-05 |
| KPS | 2018 | FarmCPU | BobWhite_049_3064 | 7A | 3,802,457 | 8.09E-07 |
| TKW | 2017 | FarmCPU | Ex_c4051_1826 | 1A | 253 | 4.30E-05 |
| TKW | 2017 | FarmCPU | BobWhite_09733_301 | 1B | 868 | 3.62E-06 |
| TKW | 2017 | FarmCPU | IAAV8042 | 5A | 2,383,891 | 8.25E-06 |
| TKW | 2017 | FarmCPU | BobWhite_rep_c49910_432 | 7B | 3,996,280 | 7.50E-08 |
| TKW | 2017 | MLM | Jagger_c3839_60 | 3D | 1,727,942 | 7.39E-06 |
| TKW | 2017 | MLM | RAC875_rep_c83934_91 | 7A | 3,886,333 | 5.30E-06 |
| TKW | 2017 | MLM | GENE_4943_470 | 7A | 3,892,017 | 4.16E-05 |
| TKW | 2017 | MLMM | BS00110286_51 | 3D | 1,727,487 | 4.98E-08 |
| TKW | 2018 | FarmCPU | Kukri_c44571_739 | 5A | 2,457,356 | 3.85E-05 |
| TKW | 2018 | FarmCPU | BS00056147_51 | 5B | 2,546,388 | 4.26E-06 |
|  |  |  |  |  |  | **(Continued)** |
| **Table S3** | |  |  |  |  | **Continued** |
| **Trait** | **Year** | **Method** | **SNPs** | **Chr** | **Position (bp)** | ***P*-Value** |
| TKW | 2018 | FarmCPU | wsnp_Ra_03949_21928888 | 6B | 3,181,171 | 2.44E-05 |
| TKW | 2018 | FarmCPU | IACX9217 | 7B | 4,211,574 | 2.67E-05 |
| TKW | 2018 | MLM | Tdurum_contig43943_56 | 1A | 90 | 1.89E-05 |
| TKW | 2018 | MLM | RAC875_25203_969 | 3B | 1,497,573 | 2.18E-05 |
| TKW | 2018 | MLM | BobWhite_rep_c67207_126 | 3B | 1,501,266 | 3.51E-06 |
| TKW | 2018 | MLM | IACX8336 | 3B | 1,502,502 | 3.57E-05 |
| TKW | 2018 | MLM | RAC875_0057_224 | 3B | 1,502,810 | 3.51E-06 |
| TKW | 2018 | MLM | RAC875_24267_258 | 3B | 1,503,428 | 2.18E-05 |
| TKW | 2018 | MLM | wsnp_Ex_07303_25979191 | 3B | 1,504,664 | 1.43E-05 |
| TKW | 2018 | MLM | wsnp_Ku_03721_21798677 | 3B | 1,508,371 | 5.40E-06 |
| TKW | 2018 | MLM | BS00079388_51 | 4A | 1,800,162 | 8.01E-06 |
| TKW | 2018 | MLM | BS00079389_51 | 4A | 1,800,391 | 1.89E-05 |
| TKW | 2018 | MLM | Kukri_006747_240 | 4A | 1,801,989 | 2.04E-05 |
| TKW | 2018 | MLM | Kukri_c67793_237 | 4A | 1,802,217 | 7.74E-06 |
| TKW | 2018 | MLM | wsnp_Ex_07338_26018247 | 4A | 1,802,674 | 2.51E-05 |
| TKW | 2018 | MLM | wsnp_Ex_21165_30292808 | 4A | 1,802,902 | 1.89E-05 |
| TKW | 2018 | MLM | wsnp_Ex_c4068_7351806 | 4A | 1,803,131 | 1.09E-05 |
| TKW | 2018 | MLM | wsnp_Ra_0022_2067517 | 4A | 1,804,272 | 2.80E-05 |
| TKW | 2018 | MLM | BS00021738_51 | 4A | 1,804,501 | 1.54E-06 |
| TKW | 2018 | MLM | Kukri_rep_005552_396 | 4A | 1,804,730 | 2.90E-05 |
| TKW | 2018 | MLM | Tdurum_contig98215_420 | 5B | 2,758,400 | 1.51E-05 |
| TKW | 2018 | MLM | Kukri_rep_c97425_164 | 7A | 3,791,938 | 2.60E-05 |
| TKW | 2018 | MLMM | Ex_07338_401 | 4A | 1,800,847 | 8.94E-08 |

KL (Kernel length); KW (Kernel width); KPS (Kernels per spike); TKW (Thousand kernel weight).

**Table S4.** Common SNPs co-detected by multiple- and single-locus GWAS methods for kernel length, kernel width, and thousand kernel weight.

| **Trait** | **Methods (1-3)** | **SNPs** | **Chr** | **Position (bp)** | ***P*-Value** |
| --- | --- | --- | --- | --- | --- |
| KL | 1,2,3 | Kukri_07961_503 | 6D | 3,491,885 | 4.03E-09 |
| KL | 2,3 | Kukri_07961_350 | 6D | 3,491,830 | 2.60E-05 |
| KW | 1,2 | Ex_07338_401 | 4A | 1,800,847 | 1.13E-07 |
| KW | 2,3 | BS00021738_51 | 4A | 1,804,501 | 4.92E-06 |
| TKW | 2,3 | BS00110286_51 | 3D | 1,727,487 | 9.27E-07 |
| TKW | 2,3 | Ex_07338_401 | 4A | 1,800,847 | 5.37E-06 |
| TKW | 1,2 | RAC875_rep_c83934_91 | 7A | 3,886,333 | 4.25E-05 |

Methods (1-3): Corresponding three single-locus GWAS methods i.e. FarmCPU, MLM, and MLMM, respectively. KL (Kernel length); KW (Kernel width); TKW (Thousand kernel weight).

**Table S5.** List of pleiotropic SNPs associated with more than one trait.

| **SNP** | **Chr** | **Position (bp)** | **Traits** |
| --- | --- | --- | --- |
| IACX938 | 4B | 2,100,182 | KL, KW |
| Excalibur_01167_1207 | 5A | 2,153,585 | TKW, KW |
| RAC875_00174_268 | 5B | 2,583,684 | TKW, KW |
| Tdurum_contig15734_221 | 7B | 4,127,623 | TKW, KL, KW |
| D_contig07330_330 | 7D | 4,292,747 | TKW, KW |

KL (Kernel length); KW (Kernel width); TKW (Thousand kernel weight).

**Table S6.** List of putative candidate genes around the peak SNPs associated with kernel width (KW) and thousand kernel weight (TKW).

| **Trait** | **QTL** | **Peak SNPs** | **Candidate genes** | **Chr** | **Physical position (bp)** | **-log P-value** | **Description** |  |
| --- | --- | --- | --- | --- | --- | --- | --- | --- |
| KW2017 | *QTLkw17_4A.10* | D_GCE8AKX01AOOSX_177 | TraesCS4B01G018900 | 4A | 13,975,408 | 5.36 | Regulatory protein NPR1 |  |
| KW2017 | *QTLkw17_4A.10* | D_GCE8AKX01AOOSX_177 | TraesCS4B01G019000 | 4A | 13,975,408 | 5.36 | Transmembrane protein 56 |  |
| KW2017 | *QTLkw17_4A.10* | D_GCE8AKX01AOOSX_177 | TraesCS4B01G019100 | 4A | 13,975,408 | 5.36 | Ras family protein |  |
| KW2017 | *QTLkw17_4A.10* | D_GCE8AKX01AOOSX_177 | TraesCS4B01G019200 | 4A | 13,975,408 | 5.36 | Trihelix transcription factor |  |
| KW2018 | *QTLkw18_3B.1* | IACX8336 | TraesCS3B01G409500 | 3B | 645,131,860 | 5.11 | Myosin-6 |  |
| KW2018 | *QTLkw18_4A.2* | wsnp_Ex_c4068_7351806 | TraesCS4A01G294400 | 4A | 595,984,826 | 5.31 | Regulatory protein NPR1 |  |
| TKW2017 | *QTLtkw17_4A.1* | Kukri_c67793_237 | TraesCS4A01G294500 | 4A | 595,821,043 | 6.05 | Transmembrane protein 56 |  |
| TKW2017 | *QTLtkw17_4A.1* | Kukri_c67793_237 | TraesCS4A01G294600 | 4A | 595,821,043 | 6.05 | Ras family protein |  |
| TKW2017 | *QTLtkw17_4A.1* | Kukri_c67793_237 | TraesCS4A01G294800 | 4A | 595,821,043 | 6.05 | Pleckstrin-like (PH) domain protein | |
| TKW2017 | *QTLtkw17_5B.1* | Tdurum_contig98215_420 | TraesCS5B01G330500 | 5B | 679,558,214 | 5.14 | Synaptotagmin, putative |  |
| TKW2017 | *QTLtkw17_5B.1* | Tdurum_contig98215_420 | TraesCS5B01G330600 | 5B | 679,558,214 | 5.14 | Synaptotagmin-2 |  |
| TKW2017 | *QTLtkw17_5B.1* | Tdurum_contig98215_420 | TraesCS5B01G330700 | 5B | 679,558,214 | 5.14 | Receptor-like kinase |  |
| TKW2017 | *QTLtkw17_5B.1* | Tdurum_contig98215_420 | TraesCS5B01G330800 | 5B | 679,558,214 | 5.14 | Photosystem I P700 chlorophyll a apoprotein A2 |  |
| TKW2017 | *QTLtkw17_5B.1* | Tdurum_contig98215_420 | TraesCS5B01G330900 | 5B | 679,558,214 | 5.14 | Strictosidine synthase |  |
| TKW2017 | *QTLtkw17_5B.1* | RAC875_rep_c83934_91 | TraesCS7A01G533900 | 7A | 712,417,045 | 6.60 | Bidirectional sugar transporter SWEET |  |
| TKW2017 | *QTLtkw17_7A.1* | RAC875_rep_c83934_91 | TraesCS7A01G534000 | 7A | 712,417,045 | 6.60 | Cytochrome P450 |  |
| TKW2017 | *QTLtkw17_7A.1* | RAC875_rep_c83934_91 | TraesCS7A01G534100 | 7A | 712,417,045 | 6.60 | Cytochrome P450 |  |
| TKW2017 | *QTLtkw17_7A.1* | RAC875_rep_c83934_91 | TraesCS7A01G534200 | 7A | 712,417,045 | 6.60 | Programmed cell death protein 2 |  |
| TKW2017 | *QTLtkw17_7A.1* | RAC875_rep_c83934_91 | TraesCS7A01G534000 | 7A | 712,417,045 | 6.60 | Cytochrome P450 |  |
| TKW2017 | *QTLtkw17_7A.1* | RAC875_rep_c83934_91 | TraesCS7A01G534100 | 7A | 712,417,045 | 6.60 | Cytochrome P450 |  |
| TKW2017 | *QTLtkw17_7A.1* | RAC875_rep_c83934_91 | TraesCS7A01G534300 | 7A | 712,417,045 | 6.60 | Coiled-coil domain-containing protein 14 |  |
| TKW2017 | *QTLtkw17_7A.1* | RAC875_rep_c83934_91 | TraesCS7A01G534200 | 7A | 712,417,045 | 6.60 | Programmed cell death protein 2 |  |
|  |  |  |  |  |  |  | **Continued** |  |
|  |  |  |  |  |  |  |  |  |
|  |  |  |  |  |  |  |  |  |
| **Table S6** |  |  |  |  |  |  | **Continued** |  |
| **Trait** | **QTL** | **Peak SNPs** | **Candidate genes** | **Chr** | **Physical position (bp)** | **-log P-value** | **Description** |  |
| TKW2017 | *QTLtkw17_7A.1* | RAC875_rep_c83934_91 | TraesCS7A01G534400 | 7A | 712,417,045 | 6.60 | Pseudouridine synthase |  |
| TKW2017 | *QTLtkw17_7A.1* | RAC875_rep_c83934_91 | TraesCS7A01G534600 | 7A | 712,417,045 | 6.60 | Pm3-like disease resistance protein |  |
| TKW2017 | *QTLtkw17_7A.1* | RAC875_rep_c83934_91 | TraesCS7A01G534700 | 7A | 712,417,045 | 6.60 | BTB/POZ domain containing protein |  |
| TKW2017 | *QTLtkw17_7A.3* | Tdurum_contig59467_433 | TraesCS7A01G538300 | 7A | 715,692,043 | 4.72 | 4-hydroxy-4-methyl-2-oxoglutarate aldolase |  |
| TKW2017 | *QTLtkw17_7A.3* | Tdurum_contig59467_433 | TraesCS7A01G538400 | 7A | 715,692,043 | 4.72 | 4-hydroxy-4-methyl-2-oxoglutarate aldolase |  |
| TKW2017 | *QTLtkw17_7A.3* | Tdurum_contig59467_433 | TraesCS7A01G538500 | 7A | 715,692,043 | 4.72 | Glyoxylate reductase / hydroxypyruvate reductase |  |
| TKW2017 | *QTLtkw17_7A.3* | Tdurum_contig59467_433 | TraesCS7A01G538600 | 7A | 715,692,043 | 4.72 | Protein Enhanced Disease Resistance 2 |  |
| TKW2017 | *QTLtkw17_7A.3* | Tdurum_contig59467_433 | TraesCS7A01G538700 | 7A | 715,692,043 | 4.72 | Hydroxysteroid dehydrogenase, putative |  |
| TKW2017 | *QTLtkw17_7A.3* | Tdurum_contig59467_433 | TraesCS7A01G538800 | 7A | 715,692,043 | 4.72 | F-box protein |  |
| TKW2018 | *QTLtkw18_3B.1* | BobWhite_rep_c67207_126 | TraesCS3B01G409500 | 3B | 645,560,691 | 6.60 | Myosin-6 |  |
| TKW2018 | *QTLtkw18_4A.1* | Ex_07338_401 | TraesCS4A01G294400 | 4A | 595,821,043 | 6.01 | Regulatory protein NPR1 |  |
| TKW2018 | *QTLtkw18_4A.1* | Ex_07338_401 | TraesCS4A01G294700 | 4A | 595,821,043 | 6.01 | Trihelix transcription factor GT-4-like protein |  |
| TKW2018 | *QTLtkw18_4A.1* | Ex_07338_401 | TraesCS4A01G294500 | 4A | 595,984,826 | 5.64 | Transmembrane protein 56 |  |
| TKW2018 | *QTLtkw18_4A.1* | Ex_07338_401 | TraesCS4A01G294600 | 4A | 595,984,826 | 5.64 | Ras family protein |  |
| TKW2018 | *QTLtkw18_4A.1* | Ex_07338_401 | TraesCS4A01G294800 | 4A | 595,984,826 | 5.64 | Ras family protein |  |
| TKW2018 | *QTLtkw18_4A.2* | BS00021738_51 | TraesCS4A01G295600 | 4A | 596,305,225 | 6.83 | Lipid transfer protein |  |
| TKW2018 | *QTLtkw18_4A.2* | BS00021738_51 | TraesCS4A01G295700 | 4A | 596,305,225 | 6.83 | Paf1 complex subunit |  |
| TKW2018 | *QTLtkw18_4A.2* | BS00021738_51 | TraesCS4A01G295800 | 4A | 596,305,225 | 6.83 | Lipid transfer protein |  |
|  |  |  |  |  |  |  | **Continued** |  |
|  |  |  |  |  |  |  |  |  |
|  |  |  |  |  |  |  |  |  |
| **Table S6** |  |  |  |  |  |  | **Continued** |  |
| **Trait** | **QTL** | **Peak SNPs** | **Candidate genes** | **Chr** | **Physical position (bp)** | **-log P-value** | **Description** |  |
| TKW2018 | *QTLtkw18_4A.2* | BS00021738_51 | TraesCS4A01G295900 | 4A | 596,305,225 | 6.83 | Nuclear transport factor 2 (NTF2) family protein with RNA binding |  |
| TKW2018 | *QTLtkw18_4A.2* | BS00021738_51 | TraesCS4A01G296000 | 4A | 596,305,225 | 6.83 | Globulin 1 |  |
| TKW2018 | *QTLtkw18_4A.2* | BS00021738_51 | TraesCS4A01G296100 | 4A | 596,305,225 | 6.83 | Globulin 1 |  |
| TKW2018 | *QTLtkw18_4A.2* | BS00021738_51 | TraesCS4A01G296200 | 4A | 596,305,225 | 6.83 | Type I inositol-1,4,5-trisphosphate 5-phosphatase CVP2 |  |
|  |  |  |  |  |  |  |  |  |
